# [3+2] Cycloaddition of Tosylmethyl Isocyanide with Styrylisoxazoles: Facile Access to Polysubstituted 3-(Isoxazol-5-yl)pyrroles

**DOI:** 10.3390/molecules22071131

**Published:** 2017-07-07

**Authors:** Xueming Zhang, Xianxiu Xu, Dawei Zhang

**Affiliations:** 1 College of Chemistry, Jilin University, Changchun 130012, China; xue15603228979@foxmail.com; 2 College of Chemistry, Chemical Engineering and Materials Science, Key Laboratory of Molecular and Nano Probes, Ministry of Education, Shandong Normal University, Jinan 250014, China; xuxx677@sdnu.edu.cn

**Keywords:** isoxazol-5-ylpyrroles, [3+2]cycloaddition, TosMIC, 3-methyl-4-nitro-5-styrylisoxazoles

## Abstract

A facile access to polysubstituted 3-(isoxazol-5-yl)pyrroles was developed through [3+2] cycloaddition of tosylmethyl isocyanide (TosMIC) and styrylisoxazoles. In the presence of KOH, various styrylisoxazoles reacted smoothly with tosylmethyl isocyanide and analogs to deliver a wide range of 3-(isoxazol-5-yl)pyrroles at ambient temperature. This transformation is operationally simple, high-yielding, and displays broad substrate scope.

## 1. Introduction

**Figure 1:**
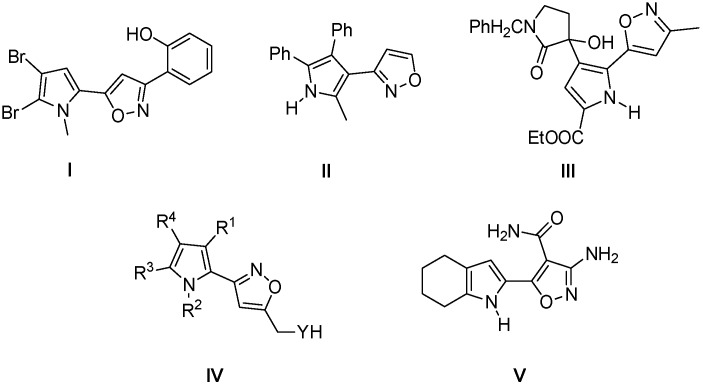
Examples of biologically active, isoxazole-substituted pyrrole derivatives.

Pyrrole derivatives are one of the most relevant heterocycles with important biological activities, which includes antitumour, antibacterial, antiviral, anti-inflammatory, antioxidative, and are also widely used in organic synthesis as key heterocycles and/or intermediates for the preparation of natural compounds and related structures, and molecular sensors [1]. In this context, isoxazole substituted pyrroles are present as the core substructure in some meaningful compounds, such as isoxazolylpyrroles **I** and **II** are inhibitors to oral and mouth cancer cell and the activators to cellular tumor antigen p53 [2,3]. Isoxazolylpyrroles **III** and **IV** are the key intermediates in the synthesis of bioactive prodiginines natural products and their congeners, and the precursors structures of phosphodiesterase inhibitors PDE-I and PDE-II, which inhibitory activity toward cyclic adenosine-3′,5′-monophosphate phosphodiesterase, respectively [4,5]. Isoxazolylpyrroles **V** is a receptor for recognition and sensing purposes in aprotic solvents [6,7]. (Figure 1).

**Scheme 1:**
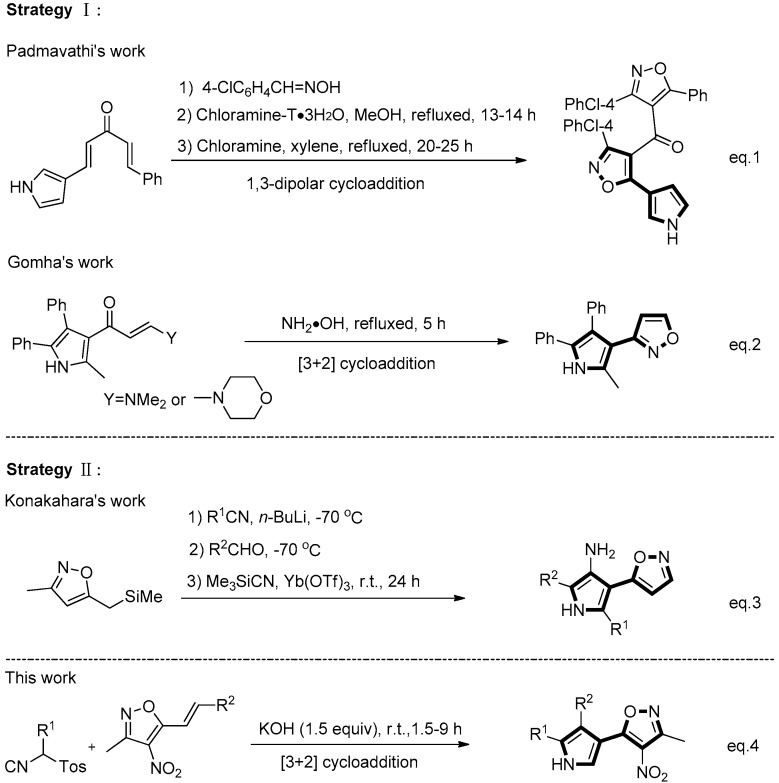
Comparison between the selected existing literature examples and this work.

In the view of the applications of isoxazole substituted pyrrole, some synthetic methods have been developed for their preparation. Among these known synthetic approaches, two main strategies are shown as follows: one is the construction of isoxazole ring from starting materials containing pyrrole ring, such as the 1,3-dipolar cycloaddition reaction of 1,5-diphenyl-1,4-pentadien-3-one with nitrile oxides in the presence of chloramine-T reported by Padmavathi et al. (Scheme 1, Equation (1)) [8] , or [3+2]-cycloadditions of enaminone and hydroxylamine hydrochloride reported by Gomha et al. (Scheme 1, Equation (2)) [3]. In contrast, another synthetic strategy is through the construction of pyrrole ring from starting materials containing isoxazole ring , including the four-component coupling reaction of a functionalized silane, a nitrile, an aldehyde, and trimethylsilylcyanide by Yb(OTf)_3_-catalyzed reported by Konakahara et al. (Scheme 1, Equation (3)) [9]. Despite these achievements, the development of novel methods for the convenient synthesis of the isoxazole substituted pyrroles is still of great interest.

In the past decades, a variety of elegant methods for the synthesis of pyrroles or oligofunctional pyrroles have been reported, including the classical Hantzsch reaction [10], the Paal-Knorr cyclization reaction [10], the van Leusen cyclization [11], and other cyclizations [11]. Among them, the [3+2] cycloaddition of tosylmethyl isocyanide with electron-deficient olefins, developed by van Leusen et al., is one of the most promising methods [12,13,14,15,16,17,18]. A wide range of electron-deficient olefins, such as α,β-unsaturated esters, ketones or nitriles, nitroolefins and styrenes, etc., are well tolerated in this reaction [19,20,21,22,23,24,25,26,27,28,29,30,31,32,33,34,35,36]. 3-Methyl-4-nitro-5-alkenylisoxazoles, developed by Adamo et al., are excellent activated olefins, which hold excellent potential for the generation of diversity [37,38,39,40]. In 2015, Adamo and co-workers reported an additional reaction of 3-methyl-4-nitro-5-alkenylisoxazoles and ethyl isocyanoacetate to give enantioenriched monoadducts; then, resulting adducts were subsequently cyclized to give 2,3-dihydropyrroles [41]. Although the stepwise synthesis of dihydropyrroles from styrylisoxazoles was developed [41], to our knowledge, the [3+2] cycloaddition reaction of styrylisoxazoles with TosMIC for the synthesis of isoxazolylpyrroles has not been reported so far. As part of our continued efforts to develop the heterocyclization of TosMIC [42,43,44,45,46,47], we report herein an expedient and convenient one-pot synthesis of isoxazole-substituted pyrrole derivatives from [3+2] cycloaddition of 3-methyl-4-nitro-5-styrylisoxazoles with TosMIC and analogs (Scheme 1, Equation (4)). Under basic conditions, various styrylisoxazoles reacted smoothly with TosMIC and analogs to deliver a wide range of polysubstituted isoxazolylpyrroles at ambient temperature.

## 2. Results and Discussion 

Initially, the reaction of TosMIC **1a** with (*E*)-5-(4-chlorostyryl)-3-methyl-4-nitroisoxazole **2b** was tested for the optimization of the reaction conditions. It was found that the reaction of **1a** and **2b** to the formation of isoxazole substituted pyrrole **3ab** in 84% yield (Table 1, entry 1) under DBU (1.5 equiv) in CH3CN at room temperature for 1 h. When the reaction time is prolonged to 6 h under the same conditions, the yield can be only improved to 87% (Table 1, entry 2). Decreasing (1.1 equiv) or increasing (1.5 equiv) the amount of TosMIC **1a** lead to almost same yield (83% and 84%) of **3ab** (Table 1, entries 3 and 4). Among the screened bases such as DBU, K_2_CO_3_, KOH, TMG, *t*-BuOK and NaOH (Table 1, entries 4–9), KOH is optimal (Table 1, entry 6). Different solvents were also surveyed, with ethanol giving comparable yield of **3ab** (Table 1, entry 10). The [3+2]-cycloaddition reaction was slower, when the reaction was performed in DMF or THF (Table 1, entries 11 and 12).


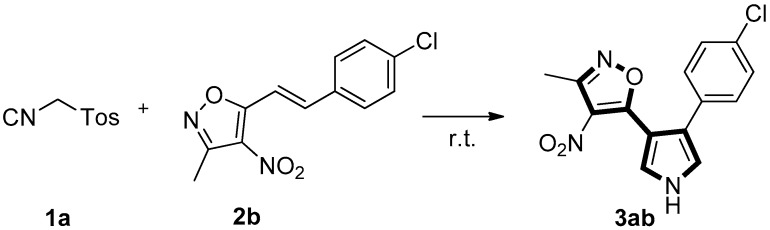


With optimal conditions in hand (Table 1, entry 6), various (*E*)-3-methyl-4-nitro-5-styrylisoxazoles **2** were explored to investigate the generality of this tandem one-pot reaction for the synthesis of **3**. The results are tabulated in Table 2. Substrates **2**, with either electron-rich or electron-deficient aryl groups, afforded the double Michael adduct **3aa**–**al** in excellent yields (Table 2, entries 1–10). Next, with the aim to explore the scope of the reaction mentioned above, a variety of (*E*)-3-methyl-4-nitro-5-(prop-1-en-1-yl)isoxazoles **2** were selected to react with TosMIC **1a** under the optimized conditions. Further experiments showed that the reaction proceeded more efficiently for the R^2^ group on (*E*)-3-methyl-4-nitro-5-(prop-1-en-1-yl)isoxazoles **2**, such as 2-furyl (**2n**), 2-thienyl (**2o**), 2-naphthyl (**2p**), and styryl (**2q**) (these groups were well tolerated) (Table 2, entries 14–17). In general, a wide range of styrylisoxazoles **2** bearing various functional groups were reacted smoothly with TosMIC **1a** under mild conditions, thus giving rise to the pyrrole products **3** in moderate to high yields.


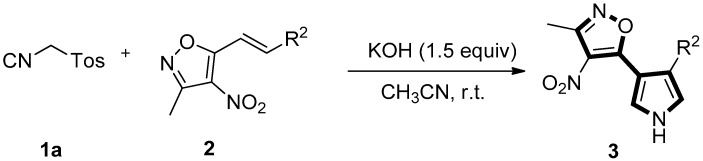


**Figure 2:**
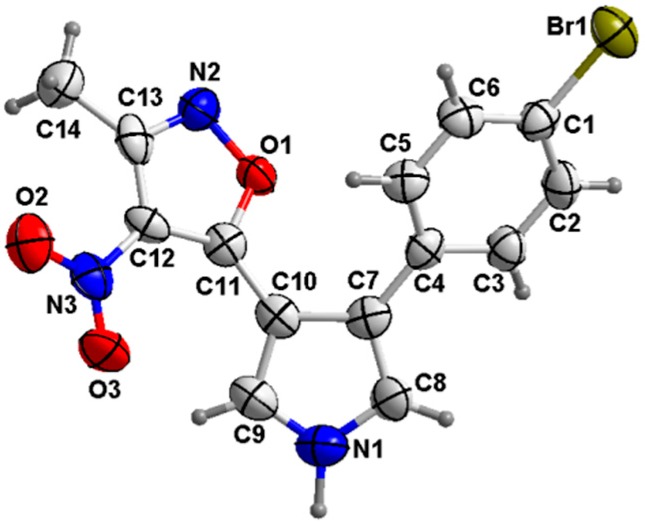
ORTEP drawing of **3ac**.

To our delight, under optimal conditions (Table 1, entry 6), further experiments showed that the R^1^ group on TosMIC **1a**, such as the ethyl (**1b**), allyl (**1c**), phenyl (**1d**), benzyl (**1e**), and *p*-methylbenzyl (**1f**) groups, also gave the corresponding trisubstituted pyrroles **3** in high yield (Table 3, entries 1–5). Therefore, a wide range of trisubstituted pyrrole derivatives were obtained under mild conditions. The configurations of pyrroles **3aa**–**fb** were assigned by NMR and high-resolution mass spectra, and the structure of **3ac** was further confirmed by the X-ray diffraction analysis (Figure 2).


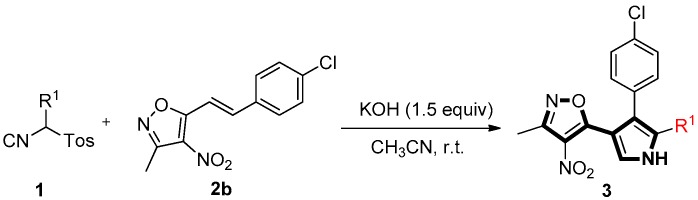


**Scheme 2:**
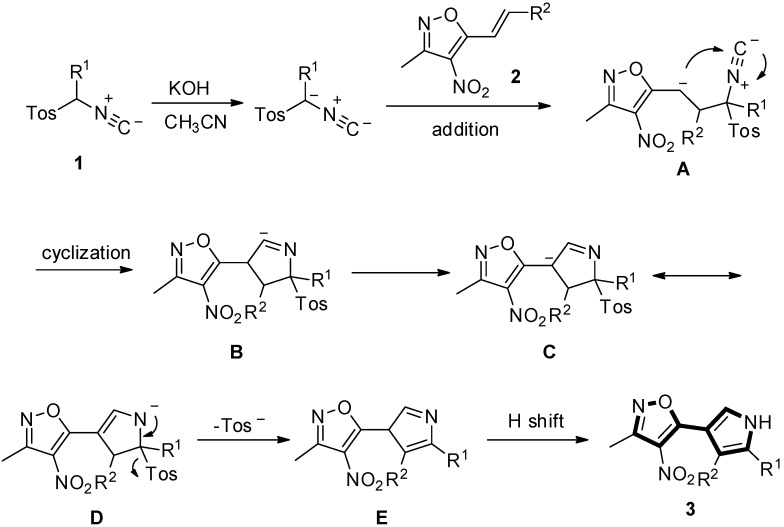
Proposed mechanism for the formation of **3**.

Generally, a stepwise mechanism rather than a concerted process is proposed in the van Leusen pyrrole synthesis from the [3+2] cycloaddition of electron-deficient olefins with TosMIC [19,20,21,22,23,24,25,26,27,28,29,30,31,32,33,34,35,36]. Thus, on the basis of the related reports [43,44,45,46,47,48] and above-stated results, a possible mechanism for the synthesis of **3** was proposed and depicted in Scheme 2. First, addition of TosMIC **1** to (*E*)-3-methyl-4-nitro-5-(prop-1-en-1-yl)isoxazole **2,** in the presence of KOH in CH_3_CN, leads to the adduct (**A**). Intramolecular cyclization of the adduct (**A**) occurs to produce the intermediate (**B**) [47]. Then, protontropic shifts, followed by the elimination of a toluenesulfinate anion to produce the intermediate (**E**) and the final hydrogen shift, deliver the 3-isoxazole-substituted pyrrole derivatives **3**.

## 3. Experimental

### 3.1. General

All reagents were commercial and used without further purification, unless otherwise indicated. Chromatography was carried on flash silica gel (300−400 mesh). All reactions were monitored by TLC, which was performed on precoated aluminum sheets of silica gel 60 (F254). Melting points were uncorrected. The ^1^H-NMR and ^13^C-NMR spectra were determined at 25 °C at 600 MHz, 150 MHz, or 125 MHz, respectively, with TMS as an internal standard. All shifts are given in ppm. High-resolution mass spectra (HRMS) were obtained using a Bruker microTOF II focus spectrometer (ESI). Crystal data was obtained by a Bruker SMART X-Ray single crystal diffractometer (Bruker, Germany). The substrates (*E*)-3-methyl-4-nitro-5-styrylisoxazoles **2** were prepared by a similar method as reported papers [49,50]. More informations can be found in the supplementary materials.

### 3.2. Synthesis of ***3aa**–**3fb***

General procedures for the synthesis of **3** (taking **3ab** as an example): to the mixture of tosylmethyl isocyanide **1a** (50.7 mg, 0.26 mmol) and (*E*)-5-(4-chlorostyryl)-3-methyl-4-nitroisoxazole **2b** (52.8 mg, 0.2 mmol) in CH_3_CN (2 mL) was added KOH (16.8 mg, 0.3 mmol), in one portion, at room temperature. The reaction mixture was stirred and monitored by TLC. After the substrate **2b** was consumed, the solvent was removed under vacuum. The crude product was subjected to column chromatography on silica gel (petroleum ether/EtOAc = 8:1) to give **3ab** (54.5 mg, 90%) as a green solid.

*3-Methyl-4-nitro-5-(4-phenyl-1H-pyrrol-3-yl)isoxazole* (**3aa**). Green solid, yield 93%, m.p. 174–176 °C. ^1^H-NMR (DMSO-*d*_6_, 600 MHz) δ 2.47 (s, 3H), 7.16 (s, 1H), 7.21 (t, *J* = 6 Hz, 3H), 7.29 (t, *J* = 7.8 Hz, 2H), 7.81 (s, 1H), 11.96 (s, 1H). ^13^C-NMR (DMSO-*d*_6_, 150 MHz) δ 12.2, 105.7, 119.8, 125.4, 126.5, 126.7, 127.6, 128.1, 128.8, 135.4, 156.5, 167.0. HRMS (ESI-TOF) *m*/*z*: Calcd. for C_14_H_12_N_3_O_3_^+^ ([M + H]^+^) 270.0873. Found: 270.0865.

*5-(4-(4-Chlorophenyl)-1H-pyrrol-3-yl)-3-methyl-4-nitroisoxazole* (**3ab**). Green solid, yield 90%, m.p. 183–185 °C. ^1^H-NMR (DMSO-*d*_6_, 600 MHz) δ 2.47 (s, 3H), 7.20 (s, 1H), 7.23 (d, *J* = 8.4 Hz, 2H), 7.34 (d, *J* = 8.4 Hz, 2H), 7.81 (s, 1H), 12.00 (s, 1H). ^13^C-NMR (DMSO-*d*_6_, 150 MHz), δ 12.2, 105.7, 120.2, 124.1, 126.8, 127.7, 128.8, 129.8, 131.4, 134.4, 156.6, 166.7. HRMS (ESI-TOF) *m*/*z*: Calcd. for C_14_H_11_ClN_3_O_3_^+^ ([M + H]^+^) 304.0483. Found: 304.0477.

*5-(4-(4-Bromophenyl)-1H-pyrrol-3-yl)-3-methyl-4-nitroisoxazole* (**3ac**). Green solid, yield 88%, m.p. 191–193 °C. ^1^H-NMR (DMSO-*d*_6_, 600 MHz) δ 2.48 (s, 3H), 7.17 (d, *J* = 8.0 Hz, 2H), 7.21 (s, 1H), 7.48 (d, *J* = 8.0 Hz, 2H), 7.81 (s, 1H), 12.01 (s, 1H). ^13^C-NMR (DMSO-*d*_6_, 150 MHz) δ 12.2, 105.6, 119.8, 120.1, 124.0, 126.6, 127.6, 130.0, 131.6, 134.8, 156.4, 166.6. HRMS (ESI-TOF) *m*/*z*: Calcd. for C_14_H_11_BrN_3_O_3_^+^ ([M + H]^+^) 347.9978. Found: 347.9978.

*3-Methyl-4-nitro-5-(4-(4-nitrophenyl)-1H-pyrrol-3-yl)isoxazole* (**3ad**). Green solid, yield 90%, m.p. 183–185 °C. ^1^H-NMR (DMSO-*d*_6_, 600 MHz) δ 2.48 (s, 3H), 7.42 (s, 1H), 7.49 (d, *J* = 9 Hz, 2H), 7.85 (s, 1H), 8.14 (d, *J* = 9 Hz, 2H), 12.20 (s, 1H). ^13^C-NMR (DMSO-*d*_6_, 150 MHz) δ 12.2, 105.9, 121.8, 123.2, 124.2, 127.3, 128.0, 128.6, 142.6, 146.0, 156.7, 166.4. HRMS (ESI-TOF) *m*/*z*: Calcd. for C_14_H_11_N_4_O_5_^+^ ([M + H]^+^) 315.0724. Found: 315.0726.

*3-Methyl-4-nitro-5-(4-(p-tolyl)-1H-pyrrol-3-yl)isoxazole* (**3ae**). Yellow solid, yield 97%, m.p. 157–159 °C. ^1^H-NMR (CDCl_3_, 600 MHz) δ 2.34 (s, 3H), 2.57 (s, 3H), 6.88 (t, *J* = 2.4 Hz, 1H), 7.13–7.16 (m, 4H), 7.84 (dd, *J*_1_ = 2.4 Hz, *J*_2_ = 0.6 Hz, 1H), 8.99 (s, 1H). ^13^C-NMR (CDCl_3_, 125 MHz) δ 12.0, 21.1, 106.8, 118.3, 125.4, 126.4, 127.4, 128.1, 129.0, 131.4, 136.5, 156.0, 166.5. HRMS (ESI-TOF) *m*/*z*: Calcd. for C_15_H_13_N_3_NaO_3_^+^ ([M + Na]^+^) 306.0849. Found: 306.0846.

*3-Methyl-4-nitro-5-(4-(m-tolyl)-1H-pyrrol-3-yl)isoxazole* (**3af**). Green solid, yield 87%, m.p. 168–170 °C. ^1^H-NMR (DMSO-*d*_6_, 600 MHz) δ 2.27 (s, 3H), 2.47 (s, 3H), 6.96 (d, *J* = 7.8 Hz, 1H), 7.03 (d, *J* = 7.8 Hz, 1H), 7.08 (s, 1H), 7.14 (t, *J* = 2.4 Hz, 1H), 7.16 (t, *J* = 7.8 Hz, 1H), 7.80 (t, *J* = 2.4 Hz, 1H), 11.95 (s, 1H). ^13^C-NMR (DMSO-*d*_6_, 150 MHz) δ 12.2, 21.6, 105.7, 119.7, 125.2, 125.4, 126.4, 127.4, 127.6, 128.6, 128.7, 135.3, 137.8, 156.4, 167.0. HRMS (ESI-TOF) *m*/*z*: Calcd. for C_15_H_14_N_3_O_3_^+^ ([M + H]^+^) 284.1030. Found: 284.1035. 

*5-(4-(3-Methoxyphenyl)-1H-pyrrol-3-yl)-3-methyl-4-nitroisoxazole* (**3ag**). Yellow solid, yield 86%, m.p. 169–171 °C. ^1^H-NMR (DMSO-*d*_6_, 600 MHz) δ 2.47 (s, 3H), 3.70 (s, 3H), 6.75–6.79 (m, 3H), 7.18–7.20 (m, 2H), 7.77–7.78 (m, 1H), 11.95 (s, 1H). ^13^C-NMR (DMSO-*d*_6_, 150 MHz) δ 12.2, 55.5, 105.7, 112.3, 113.5, 119.9, 120.4, 125.2, 126.4, 127.7, 129.9, 136.7, 156.5, 159.7, 167.0. HRMS (ESI-TOF) *m*/*z*: Calcd. for C_15_H_13_N_3_NaO_4_^+^ ([M + Na]^+^) 322.0798. Found: 322.0795.

*5-(4-(3-Chlorophenyl)-1H-pyrrol-3-yl)-3-methyl-4-nitroisoxazole* (**3ah**). Paleyellow solid, yield 86%, m.p.163–165 °C. ^1^H-NMR (DMSO-*d*_6_, 600 MHz) δ 2.47 (s, 3H), 7.13 (d, *J* = 7.8 Hz, 1H), 7.26–7.32 (m, 4H), 7.83 (t, *J* = 2.4 Hz, 1H), 12.05 (s, 1H). ^13^C-NMR (DMSO-*d*_6_, 150 MHz) δ 12.2, 105.7, 120.6, 123.7, 126.5, 126.7, 126.8, 127.6, 127.7, 130.6, 133.5, 137.6, 156.5, 166.6. HRMS (ESI-TOF) *m*/*z*: Calcd. for C_14_H_11_ClN_3_O_3_^+^ ([M + H]^+^) 304.0483. Found: 304.0474.

*3-Methyl-4-nitro-5-(4-(o-tolyl)-1H-pyrrol-3-yl)isoxazole* (**3ai**). Yellow solid, yield 92%, m.p. 185–187 °C. ^1^H-NMR (CDCl_3_, 600 MHz) δ 2.11 (s, 3H), 2.52 (s, 3H), 6.79–6.80 (m, 1H), 7.16–7.17 (m, 2H), 7.21–7.24 (m, 2H), 8.11–8.12 (m, 1H), 8.95 (s, 1H). ^13^C-NMR (CDCl_3_, 125 MHz) δ 12.1, 20.1, 108.5, 118.9, 125.4, 125.4, 125.4, 126.7, 127.5, 129.9, 130.4, 134.2, 136.9, 155.9, 166.2. HRMS (ESI-TOF) *m*/*z*: Calcd. for C_15_H_13_N_3_NaO_3_^+^ ([M + Na]^+^) 306.0849. Found: 306.0854.

*5-(4-(2-Chlorophenyl)-1H-pyrrol-3-yl)-3-methyl-4-nitroisoxazole* (**3aj**). Green solid, yield 89%, m.p. 165–167 °C. ^1^H-NMR (CDCl_3_, 600 MHz) δ 2.54 (s, 3H), 6.90 (t, *J* = 2.4 Hz, 1H), 7.24–7.27 (m, 2H), 7.31 (dd, *J*_1_ = 3.6 Hz, *J*_2_ = 2.4 Hz, 1H), 7.4 (dd, *J*_1_ = 3.6 Hz, *J*_2_ = 2.4 Hz, 1H), 8.11 (dd, *J*_1_ = 2.4 Hz, *J*_2_ = 0.6 Hz, 1H), 8.98 (s, 1H). ^13^C-NMR (CDCl_3_, 125 MHz) δ 12.1, 108.6, 119.6, 123.3, 125.4, 126.6, 128.7, 129.5, 131.6, 133.6, 133.9, 156.0, 166.1. HRMS (ESI-TOF) *m*/*z*: Calcd. for C_14_H_11_ClN_3_O_3_^+^ ([M + H]^+^) 304.0483. Found: 304.0482.

*5-(4-(2,3-Dichlorophenyl)-1H-pyrrol-3-yl)-3-methyl-4-nitroisoxazole* (**3ak**). Green solid, yield 57%, m.p. 177–179 °C. ^1^H-NMR (CDCl_3_, 600 MHz) δ 2.54 (s, 3H), 6.92 (s, 1H), 7.20 (d, *J* = 8.8 Hz, 2H), 7.43 (d, *J* = 8.8 Hz, 1H), 8.15 (s, 1H), 8.95 (s, 1H). ^13^C-NMR (CDCl_3_, 125 MHz) δ 12.1, 108.7, 119.7, 123.2, 125.5, 126.9, 129.7, 129.9, 132.5, 133.3, 136.0, 156.0, 165.7. HRMS (ESI-TOF) *m*/*z*: Calcd. for C_14_H_10_Cl_2_N_3_O_3_^+^ ([M + H]^+^) 338.0094. Found: 338.0080.

*5-(4-(3,4-Dichlorophenyl)-1H-pyrrol-3-yl)-3-methyl-4-nitroisoxazole* (**3al**). Green solid, yield 78%, m.p. 174–176 °C. ^1^H-NMR (CDCl_3_, 600 MHz) δ 2.58 (s, 3H), 6.95 (t, *J* = 2.4 Hz, 1H), 7.06 (dd, *J*_1_ = 1.8 Hz, *J*_2_ = 6.6 Hz, 1H), 7.38–7.39 (m, 2H), 7.94–7.95 (m, 1H), 8.92 (s, 1H). ^13^C-NMR (CDCl_3_, 125 MHz) δ 12.1, 107.1, 118.9, 124.3, 125.8, 127.8, 130.1, 130.2, 131.0, 132.3, 134.5, 156.2, 165.6. HRMS (ESI-TOF) *m*/*z*: Calcd. for C_14_H_10_Cl_2_N_3_O_3_^+^ ([M + H]^+^) 338.0094. Found: 338.0080.

*5-(4-(2,5-Dimethoxyphenyl)-1H-pyrrol-3-yl)-3-methyl-4-nitroisoxazole* (**3am**). Yellow solid, yield 86%, m.p. 172–174 °C. ^1^H-NMR (DMSO-*d*_6_, 600 MHz) δ 2.46 (s, 3H), 3.33 (s, 3H), 3.71 (s, 3H), 6.78–6.80 (m, 1H), 6.82–6.84 (m, 2H), 7.08 (t, *J* = 2.4 Hz, 1H), 7.80 (t, *J* = 3 Hz, 1H), 11.89 (s, 1H). ^13^C-NMR (DMSO-*d*_6_, 150 MHz) δ 12.1, 55.7, 55.8, 107.3, 112.4, 112.8, 116.3, 120.4, 121.6, 125.2, 125.7, 126.6, 150.5, 153.5, 156.0, 168.0. HRMS (ESI-TOF) *m*/*z*: Calcd. for C_16_H_16_N_3_O_5_^+^ ([M + H]^+^) 330.1084. Found: 330.1095.

*5-(4-(Furan-2-yl)-1H-pyrrol-3-yl)-3-methyl-4-nitroisoxazole* (**3an**). Yellow solid, yield 84%, m.p. 148–150 °C. ^1^H-NMR (DMSO-*d*_6_, 600 MHz) δ 2.51 (s, 3H), 6.34 (d, *J* = 3 Hz, 1H), 6.45 (dd, *J*_1_ = 1.8 Hz, *J*_2_ = 1.2 Hz, 1H), 7.31 (d, *J* = 1.8 Hz, 1H), 7.52 (s, 1H), 7.73 (s, 1H), 12.02 (s, 1H). ^13^C-NMR (DMSO-*d*_6_, 150 MHz) δ 12.1, 104.8, 105.5, 111.8, 115.1, 119.5, 125.9, 128.0, 141.9, 149.3, 156.5, 166.5. HRMS (ESI-TOF) *m*/*z*: Calcd. for C_12_H_10_N_3_O_4_^+^ ([M + H]^+^) 260.0666. Found: 260.0669. 

*3-Methyl-4-nitro-5-(4-(thiophen-2-yl)-1H-pyrrol-3-yl)isoxazole* (**3ao**). Yellow solid, yield 81%, m.p. 115–117 °C. ^1^H-NMR (DMSO-*d*_6_, 600 MHz) δ 2.49 (s, 3H), 6.90 (d, *J* = 3 Hz, 1H), 6.99 (dd, *J* = 3.6 Hz, *J* = 1.2 Hz, 1H), 7.21 (t, *J* = 2.4 Hz, 1H), 7.37 (d, *J* = 5.4 Hz, 1H), 7.76 (t, *J* = 2.4 Hz, 1H), 12.01 (s, 1H). ^13^C-NMR (DMSO-*d*_6_, 150 MHz) δ 12.1, 105.8, 117.9, 120.2, 124.8, 124.9, 126.2, 128.0, 128.0, 136.6, 156.5, 166.5. HRMS (ESI-TOF) *m*/*z*: Calcd. for C_12_H_10_N_3_O_3_S^+^ ([M + H]^+^) 276.0437. Found: 276.0446.

*3-Methyl-5-(4-(naphthalen-2-yl)-1H-pyrrol-3-yl)-4-nitroisoxazole* (**3ap**). Yellow solid, yield 90%, m.p. 210–212 °C. ^1^H-NMR (DMSO-*d*_6_, 600 MHz) δ 2.49 (s, 3H), 7.32 (s, 1H), 7.39 (d, *J* = 8.4 Hz, 1H), 7.46–7.48 (m, 2H), 7.79 (s, 1H), 7.84 (d, *J* = 7.2 Hz, 2H), 7.88 (d, *J* = 7.8 Hz, 1H), 7.90 (s, 1H), 12.07 (s, 1H). ^13^C-NMR (DMSO-*d*_6_, 150 MHz) δ 12.2, 105.9, 120.3, 125.3, 125.8, 126.0, 126.6, 126.7, 127.2, 127.6, 127.9, 128.1, 128.2, 132.1, 133.0, 133.7, 156.5, 166.9. HRMS (ESI-TOF) *m*/*z*: Calcd. for C_18_H_14_N_3_O_3_^+^ ([M + H]^+^) 320.1030. Found: 320.1027.

*(E)-3-Methyl-4-nitro-5-(4-styryl-1H-pyrrol-3-yl)isoxazole* (**3aq**). Orange solid, yield 82%, m.p. 177–179 °C. ^1^H-NMR (CDCl_3_, 600 MHz) δ 2.62 (s, 3H), 6.87 (d, *J* = 16.2 Hz, 1H), 7.17 (s, 1H), 7.24 (t, *J* = 7.2 Hz, 1H), 7.33 (m, 2H), 7.41 (d, *J* = 16.2 Hz, 1H), 7.47 (d, *J* = 7.8 Hz, 2H), 8.10–8.11 (m,1H), 8.83 (s, 1H). ^13^C-NMR (CDCl_3_, 125 MHz) δ 12.2, 107.6, 116.5, 120.8, 124.0, 126.1, 126.3, 127.4, 128.6, 128.9, 137.4, 156.3, 166.4. HRMS (ESI-TOF) *m*/*z*: Calcd. for C_16_H_14_N_3_O_3_^+^ ([M + H]^+^) 296.1030. Found: 296.1028.

*5-(4-(4-Chlorophenyl)-5-ethyl-1H-pyrrol-3-yl)-3-methyl-4-nitroisoxazole* (**3bb**). Green solid, yield 67%, m.p. 182–184 °C. ^1^H-NMR (CDCl_3_, 600 MHz) δ 1.19 (t, *J* = 7.8 Hz, 3H), 2.52 (s, 3H), 2.60 (dd, *J_1_* = 7.8 Hz, *J_2_* = 7.2 Hz, 2H), 7.13 (m, 2H), 7.32 (m, 2H), 7.94 (d, *J* = 1.8 Hz, 1H), 8.66 (s, 1H). ^13^C-NMR (CDCl_3_, 125 MHz) δ 12.1, 14.1, 18.9, 29.7, 108.1, 120.3, 123.9, 128.3, 131.2, 132.8, 133.2, 133.4, 156.0, 166.1. HRMS (ESI-TOF) *m*/*z*: Calcd. for C_16_H_15_ClN_3_O_3_^+^ ([M + H]^+^) 332.0796. Found: 332.0799.

*5-(5-Allyl-4-(4-chlorophenyl)-1H-pyrrol-3-yl)-3-methyl-4-nitroisoxazole* (**3cb**). Green solid, yield 56%, m.p. 171–173 °C. ^1^H-NMR (CDCl_3_, 600 MHz) δ 2.52 (d, 3H), 3.33 (d, *J* = 6 Hz, 2H), 5.18 (m, 2H), 5.89 (m, 1H), 7.13 (d, *J* = 8.4 Hz, 2H), 7.32 (d, *J* = 8.4 Hz, 2H), 7.95 (d, *J* = 3 Hz, 1H), 8.58 (s, 1H). 13C-NMR (CDCl_3_, 125 MHz) δ 12.1, 30.1, 108.2, 118.0, 121.2, 124.2, 128.4, 129.0, 131.1, 132.8, 132.9, 134.5, 156.0, 166.0. HRMS (ESI-TOF) *m*/*z*: Calcd. for C_17_H_15_ClN_3_O_3_^+^ ([M + H]^+^) 344.0796. Found: 344.0797.

*5-(4-(4-Chlorophenyl)-5-phenyl-1H-pyrrol-3-yl)-3-methyl-4-nitroisoxazole* (**3db**). Green solid, yield 81%, m.p. 257–259 °C. ^1^H-NMR (DMSO-*d*_6_, 600 MHz) δ 2.44 (s, 3H), 7.14 (d, *J* = 8.5 Hz, 2H), 7.22–7.25 (m, 3H), 7.30–7.33 (m, 4H), 7.98 (d, *J* = 2 Hz, 1H), 12.40 (s, 1H). ^13^C-NMR (DMSO-*d*_6_, 150 MHz) δ 12.2, 108.5, 120.5, 126.3, 127.7, 127.8, 128.1, 128.8, 129.1, 131.2, 131.7, 132.0, 132.3, 134.2, 156.3, 166.2. HRMS (ESI-TOF) *m*/*z*: Calcd. for C_20_H_15_ClN_3_O_3_^+^ ([M + H]^+^) 380.0796. Found: 380.0792.

*5-(5-Benzyl-4-(4-chlorophenyl)-1H-pyrrol-3-yl)-3-methyl-4-nitroisoxazole* (**3eb**). Green solid, yield 78%, m.p. 197–199 °C. ^1^H-NMR (CDCl_3_, 600 MHz) δ 2.52 (s, 3H), 3.93 (s, 2H), 7.14 (d, *J* = 7.2 Hz, 2H), 7.18–7.19 (m, 2H), 7.26 (d, *J* = 14.4 Hz, 1H), 7.31–7.34 (m, 4H), 7.91 (d, *J* = 3 Hz, 1H), 8.43 (s, 1H). ^13^C-NMR (CDCl_3_, 125 MHz) δ 12.6, 31.8, 108.2, 121.6, 124.5, 127.0, 128.5, 128.6, 129.0, 130.2, 131.2, 132.9, 133.0, 137.9, 156.0, 166.0. HRMS (ESI-TOF) *m*/*z*: Calcd. for C_21_H_17_ClN_3_O_3_^+^ ([M + H]^+^) 394.0953. Found: 394.0950.

*5-(4-(4-Chlorophenyl)-5-(4-methylbenzyl)-1H-pyrrol-3-yl)-3-methyl-4-nitroisoxazole* (**3fb**). Green solid, yield 83%, m.p. 167–169 °C. ^1^H-NMR (CDCl_3_, 600 MHz) δ 2.33 (s, 3H), 2.52 (s, 3H), 3.88 (s, 2H), 7.03 (d, *J* = 7.8 Hz, 2H), 7.13 (d, *J* = 7.8 Hz , 2H), 7.19 (d, *J* = 8.4 Hz, 2H), 7.33 (d, *J* = 7.8 Hz, 2H), 7.90 (d, *J* = 3 Hz, 1H), 8.46 (s, 1H). ^13^C-NMR (CDCl_3_, 125 MHz) δ 12.1, 20.9, 31.3, 108.1, 121.4, 124.4, 127.1, 128.4, 129.7, 130.6, 131.2, 132.9, 134.7, 136.7, 155.9, 166.00. HRMS (ESI-TOF) *m*/*z*: Calcd. for C_22_H_19_ClN_3_O_3_^+^ ([M + H]^+^) 408.1109. Found: 408.1103.

### 3.3. Crystal Structure Determination

Single crystal of **3ac**, suitable for X-ray diffraction analysis, was obtained by slow evaporation of its solution in petroleum ether-EtOAc (8:1, *v*/*v*) at room temperature. Selected light green single crystal of **3ac** was mounted on glass fibers. The intensity data were measured at 293 K on a Bruker SMART APEXII CCD; cell refinement: *SAINT* (Bruker, Billerica, MA, USA 2007); data reduction: *SAINT*; program(s) used to solve structure: *SHELXS97* [51]; program(s) used to refine structure: *SHELXL97* [51]; molecular graphics: *SHELXTL* [51]; software used to prepare material for publication: *SHELXTL* [51]. Crystallographic data for the structures **3ac** have been deposited in the Cambridge Crystallography Data Centre (CCDC No. 1552332).

## 4. Conclusions

In summary, we have developed an efficient tandem one-pot synthesis of the isoxazole-substituted pyrrole derivatives via [3+2] cycloaddition of TosMIC and analogs with various styrylisoxazoles. This reaction features high efficiency, mild reaction conditions, broad substrate scope, and readily available substrates. Further investigations on the bicyclization strategy of activated isocyanides for the divergent synthesis of complex architecture are currently underway in our laboratory.

## Figures and Tables

**Table 1:** Optimization of the reaction conditions.

Entry	1a:2b	Base (equiv)	Solvent	Time (h)	Yield (%) ^a^
1	1.3:1	DBU (1.5)	CH_3_CN	1.0	84
2	1.3:1	DBU (1.5)	CH_3_CN	6.0	87
3	1.1:1	DBU (1.5)	CH_3_CN	1.5	83
4	1.5:1	DBU (1.5)	CH_3_CN	1.5	84
5	1.3:1	K_2_CO_3_ (1.5)	CH_3_CN	8.0	82
**6**	**1.3:1**	**KOH (1.5)**	**CH_3_CN**	**2.5**	**90**
7	1.3:1	TMG (1.5)	CH_3_CN	0.5	82
8	1.3:1	*t*-BuOK (1.5)	CH_3_CN	1.5	77
9	1.3:1	NaOH (1.5)	CH_3_CN	1.0	82
10	1.3:1	KOH (1.5)	EtOH	2.0	80
11	1.3:1	KOH (1.5)	DMF	1.5	63
12	1.3:1	KOH (1.5)	THF	2.0	70

^a^ Yield of isolated product **3ab**.

**Table 2:** Synthesis of 3-isoxazole bisubstituted pyrrole derivatives **1**–**17**.

Entry	R^2^	Time (h)	3	Yield (%) ^a^
1	Ph	4.0	**aa**	93
2	4-ClC_6_H_4_	2.5	**ab**	90
3	4-BrC_6_H_4_	5.5	**ac**	88
4	4-NO_2_C_6_H_4_	4.5	**ad**	90
5	4-CH_3_C_6_H_4_	3.5	**ae**	97
6	3-CH_3_C_6_H_4_	4.0	**af**	87
7	3-OCH_3_C_6_H_4_	1.5	**ag**	86
8	3-ClC_6_H_4_	3.5	**ah**	86
9	2-CH_3_C_6_H_4_	1.5	**ai**	92
10	2-ClC_6_H_4_	5.0	**aj**	89
11	2,3-ClC_6_H_3_	3.5	**ak**	57
12	3,4-Cl_2_C_6_H_3_	4.5	**al**	78
13	2,5-(OCH_3_)_2_C_6_H_3_	4.0	**am**	86
14	2-furyl	3.5	**an**	84
15	2-thienyl	3.5	**ao**	81
16	2-naphthyl	5.0	**ap**	90
17	C_6_H_5_CH=CH	3.0	**aq**	82

^a^ Yields of isolated product.

**Table 3:** Synthesis of 3-isoxazole trisubstituted pyrrole derivatives **1**–**5**.

Entry	R^1^	Time (h)	3	Yield (%) ^a^
1	CH_3_CH_2_	8.0	**bb**	67
2	allyl	9.0	**cb**	56
3	C_6_H_5_	4.0	**db**	81
4	C_6_H_5_CH_2_	7.0	**eb**	78
5	4-CH_3_C_6_H_4_CH_2_	5.0	**fb**	83

^a^ Yields of isolated product.

## Supplementary Materials

Supplementary data associated with this article can be found in the SI.
