# Supplementary material for: [3+2] Cycloaddition of Tosylmethyl Isocyanide with Styrylisoxazoles: Facile Access to Polysubstituted 3-(Isoxazol-5-yl)pyrroles
Source: Molecules. 2017 Jul 7;22(7):1131. doi: 10.3390/molecules22071131 (PMC6152356; doi:10.3390/molecules22071131)
Supplement: Supplementary file 1 [file molecules-22-01131-s001.pdf]

Supporting Information

[3+2] Cycloaddition of Tosylmethyl Isocyanide with Styrylisoxazoles: Facile Access to Polysubstituted 3-(Isoxazol-5-yl)pyrroles

Xueming Zhang[1], Xianxiu Xu[2] and Dawei Zhang[1,*]

[1] College of Chemistry, Jilin University, Changchun 130012, China;

[2] College of Chemistry, Chemical Engineering and Materials Science, Key Laboratory of Molecular and Nano Probes, Ministry of Education, Shandong Normal University, Jinan 250014, China;

* Correspondence: z_dw@jlu.edu.cn; Tel.: +86-431-878-36471

Contents

I. Copies of ^{1}H NMR and ^{13}C NMR spectra of compounds **3aa-3fb ……………...……..……. ..**S2

II. Copies of NOE-NMR spectra of compounds **3aq …………………...…………..…..…. .**S24

III. Crystal data and structural refinement for **3ac…………………...…………..……..…..……**S25

I. Copies of ^{1}H NMR and ^{13}C NMR spectra of compounds **3aa-3fb**

^{1}H NMR and ^{13}C NMR of **3aa**

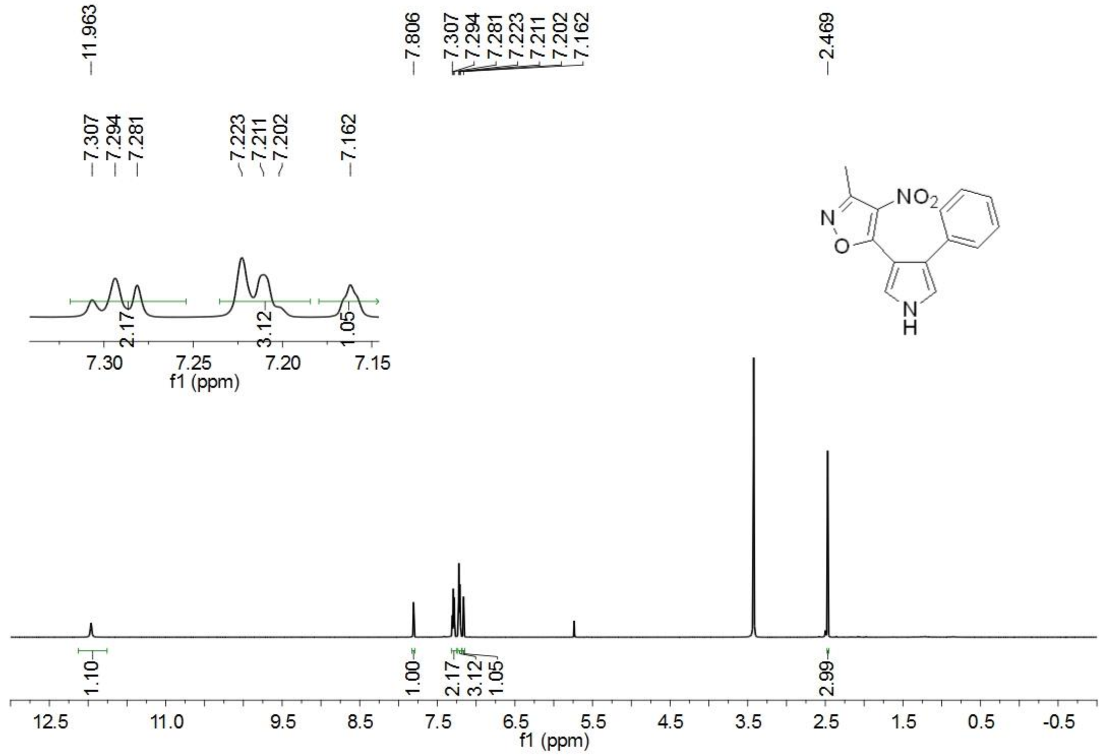

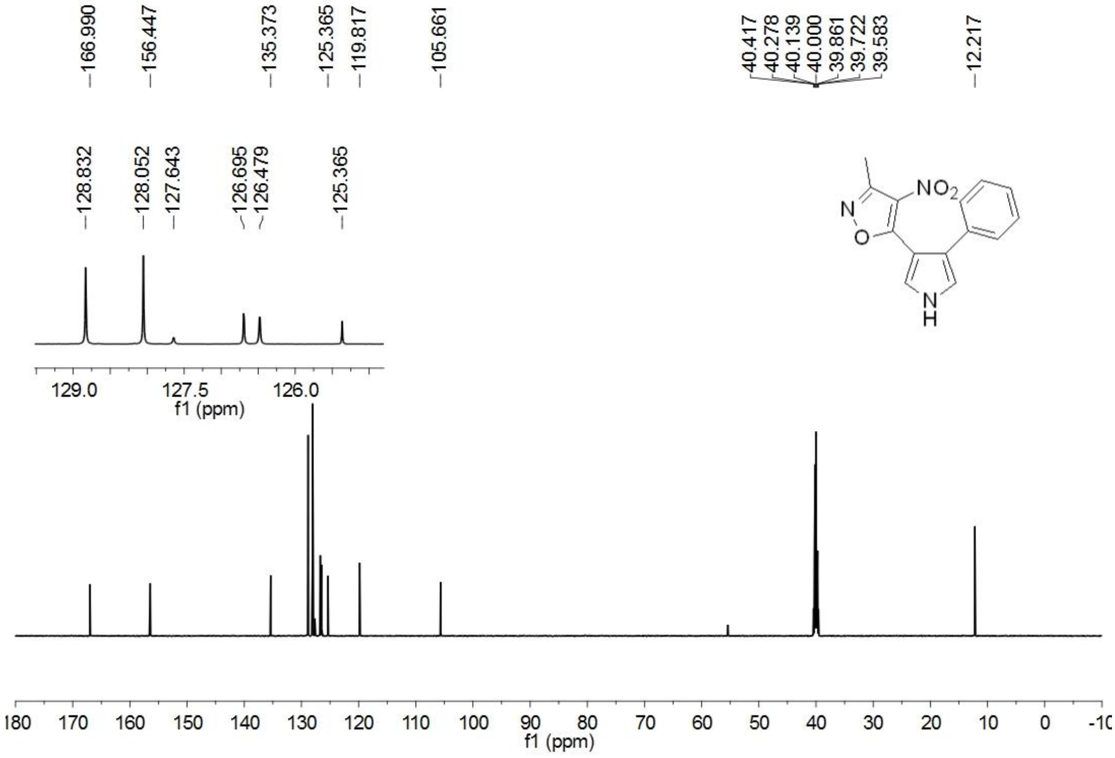

^{1}H NMR and ^{13}C NMR of **3ab**

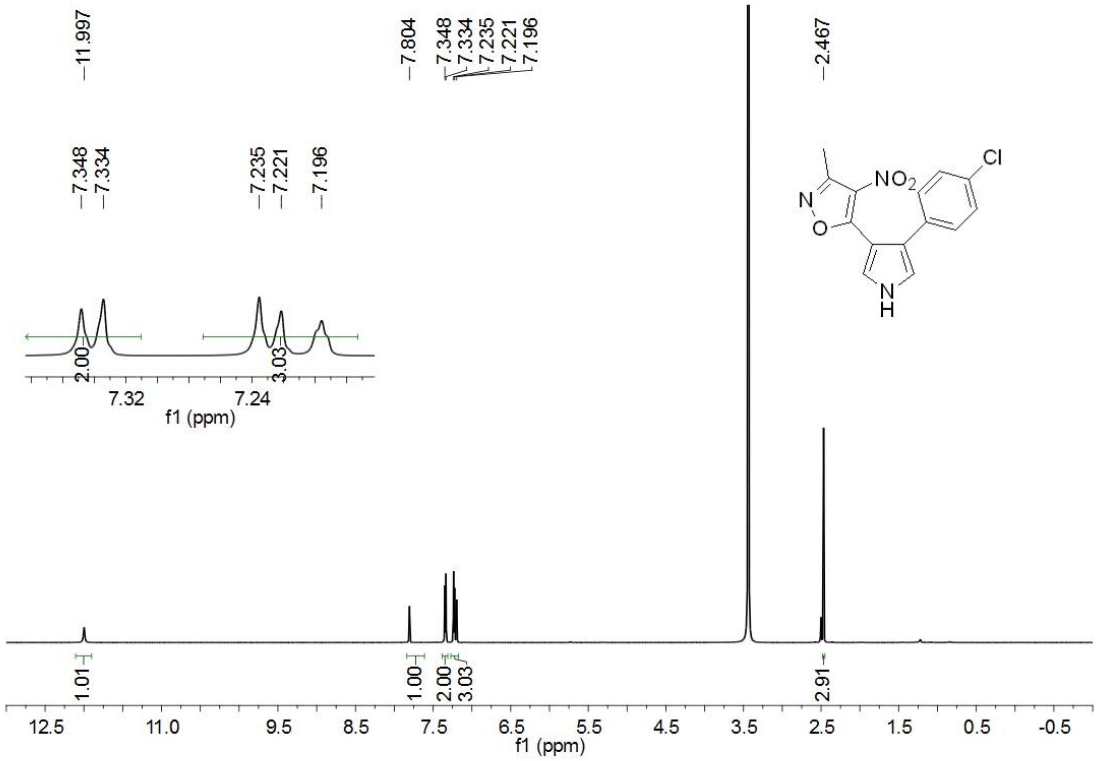

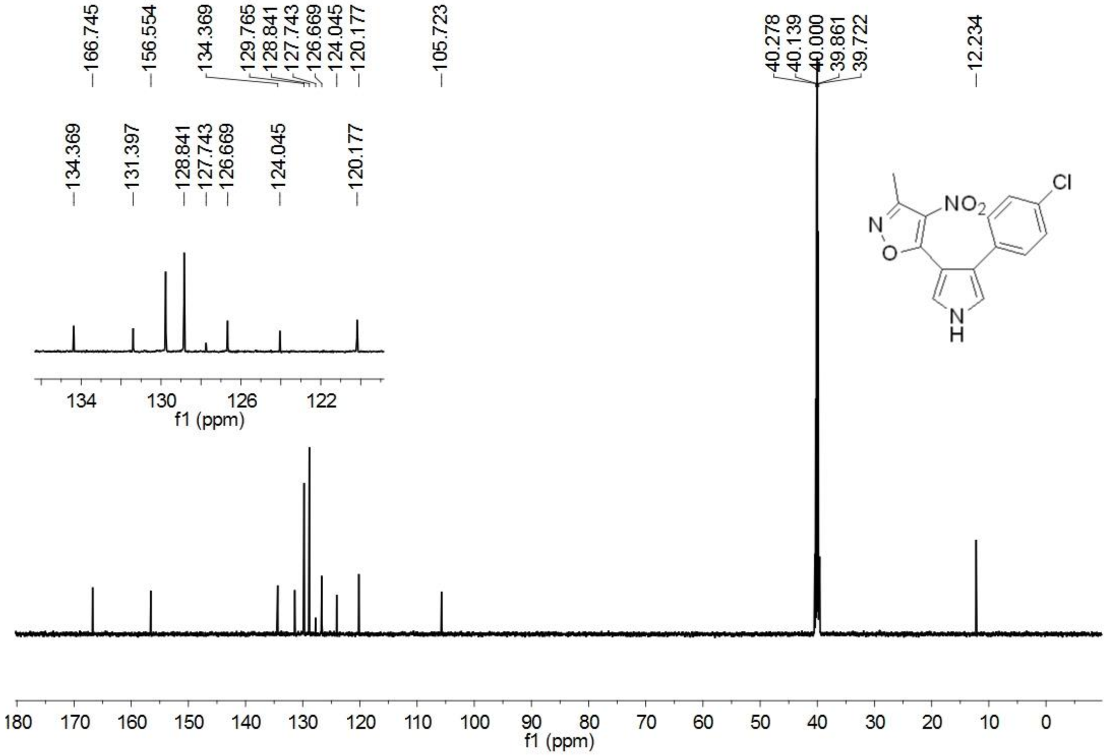

^{1}H NMR and ^{13}C NMR of **3ac**

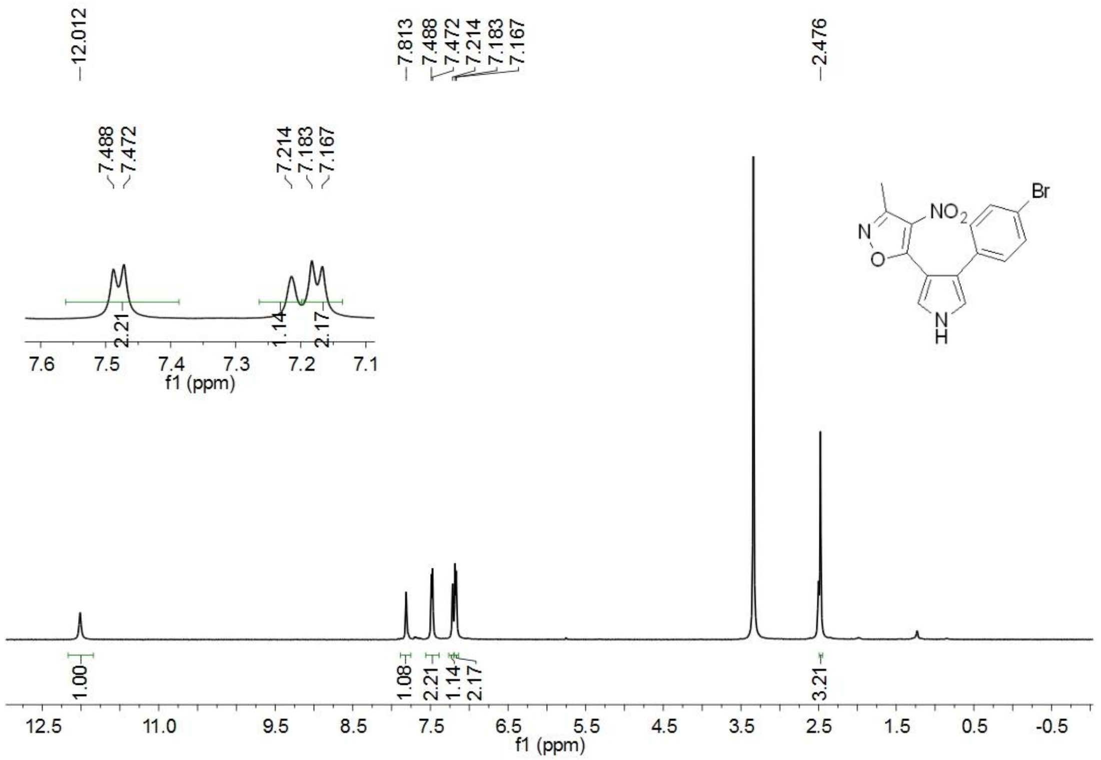

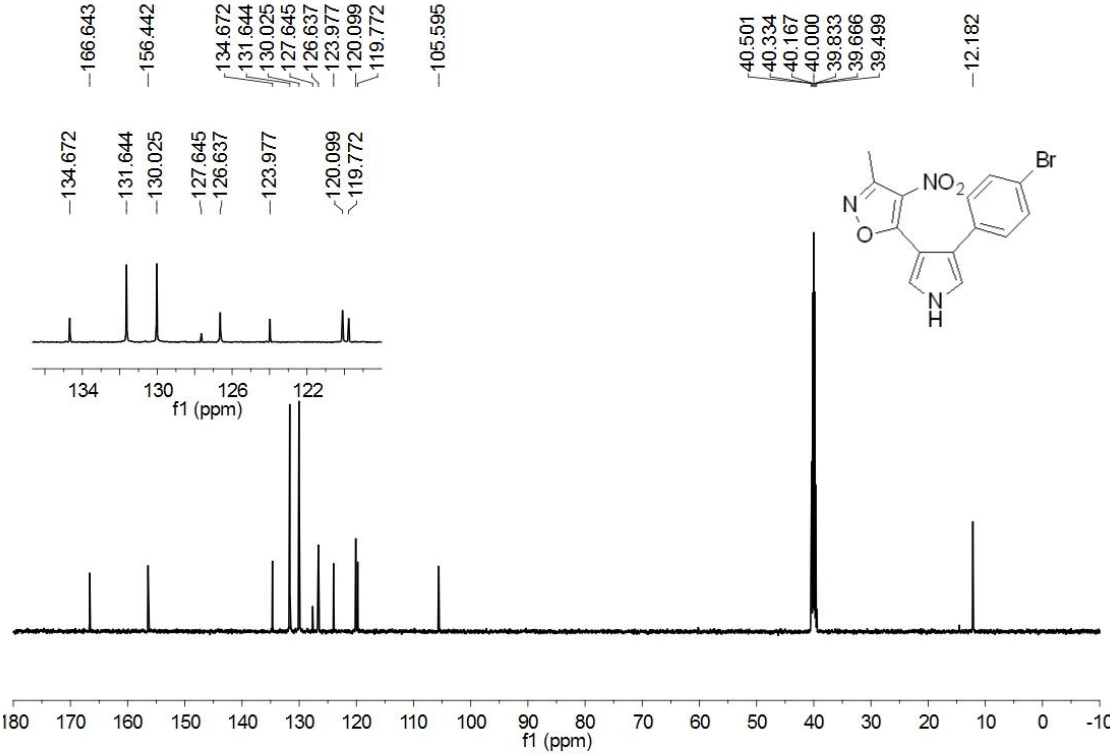

^{1}H NMR and ^{13}C NMR of **3ad**

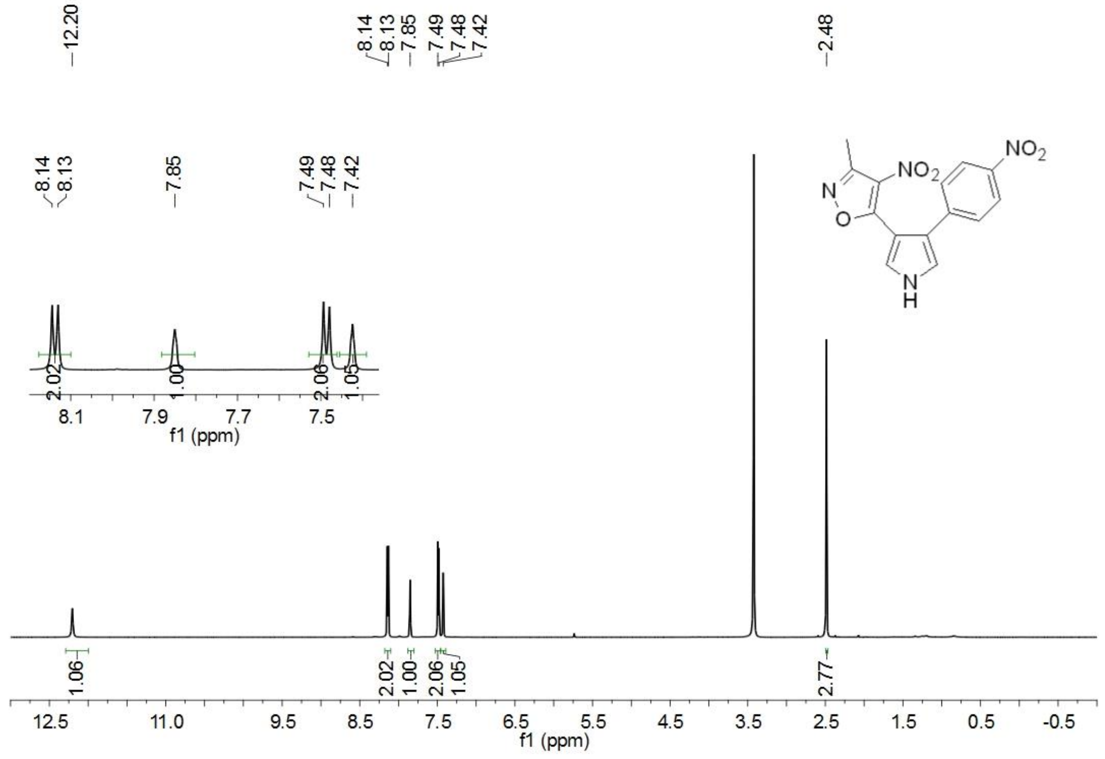

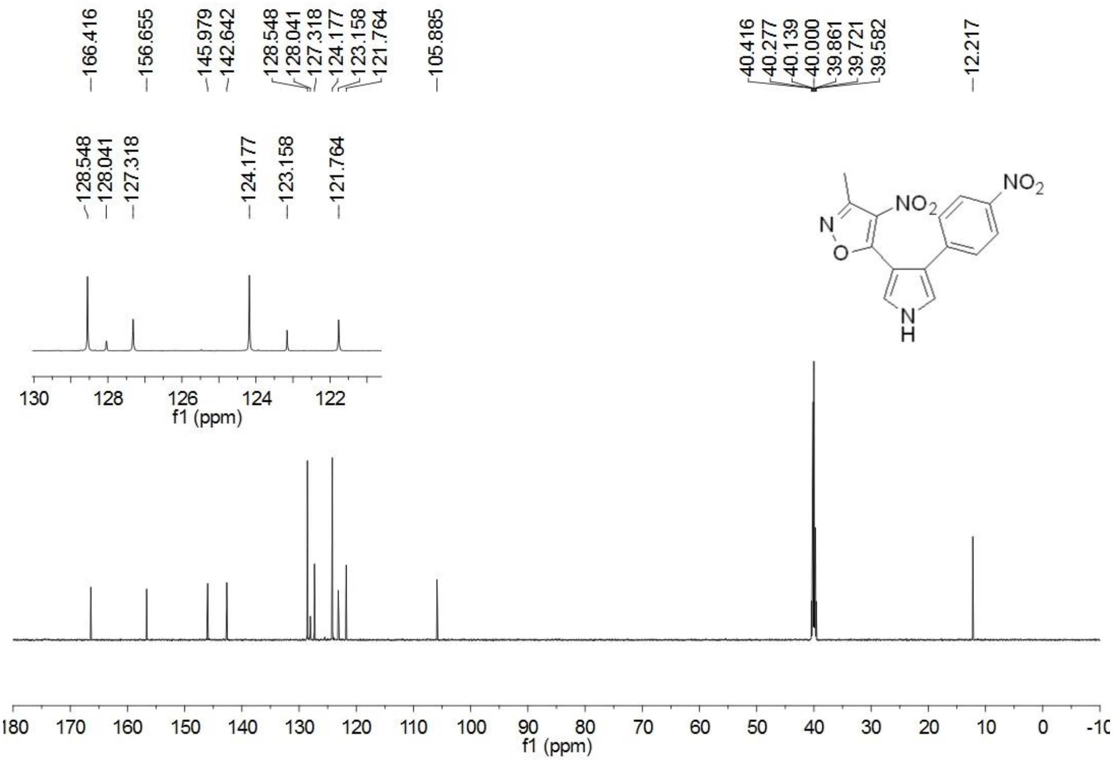

^{1}H NMR and ^{13}C NMR of **3ae**

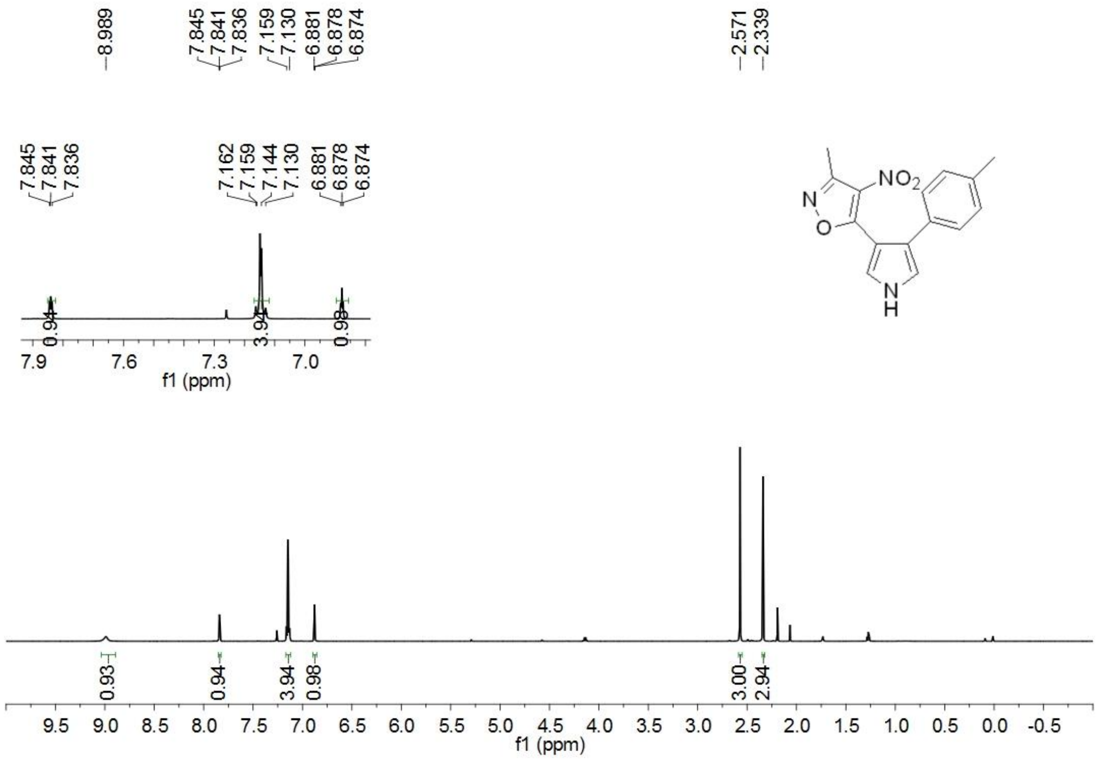

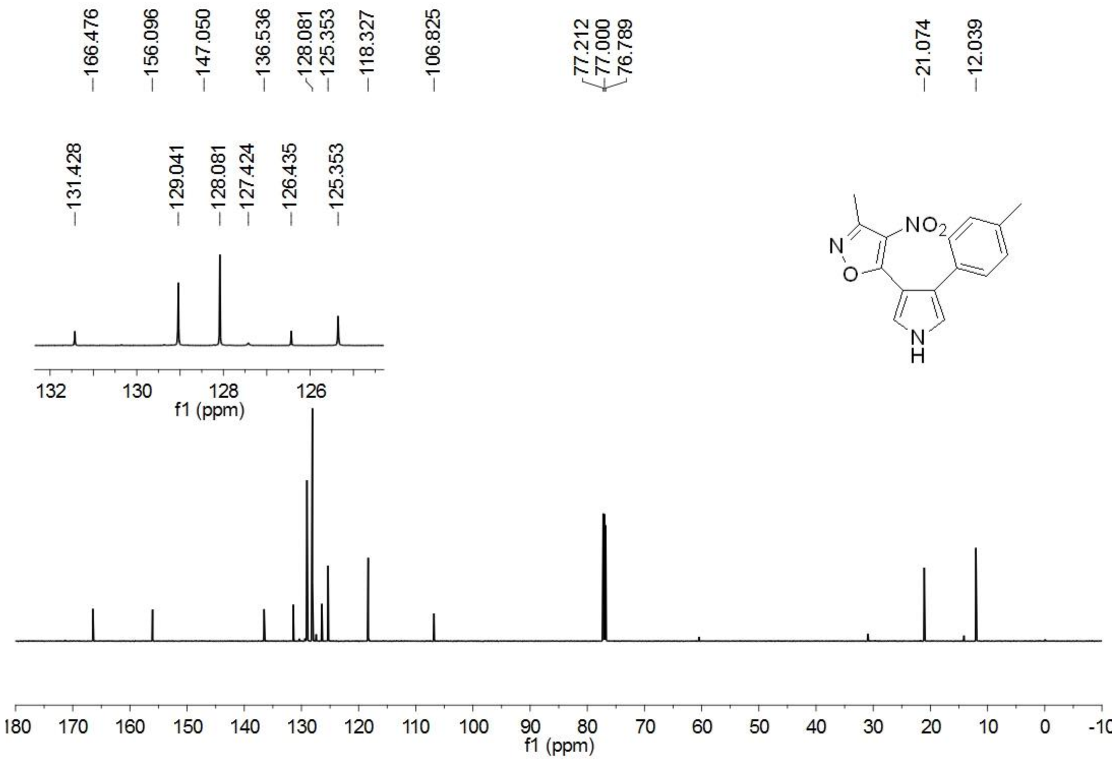

^{1}H NMR and ^{13}C NMR of **3af**

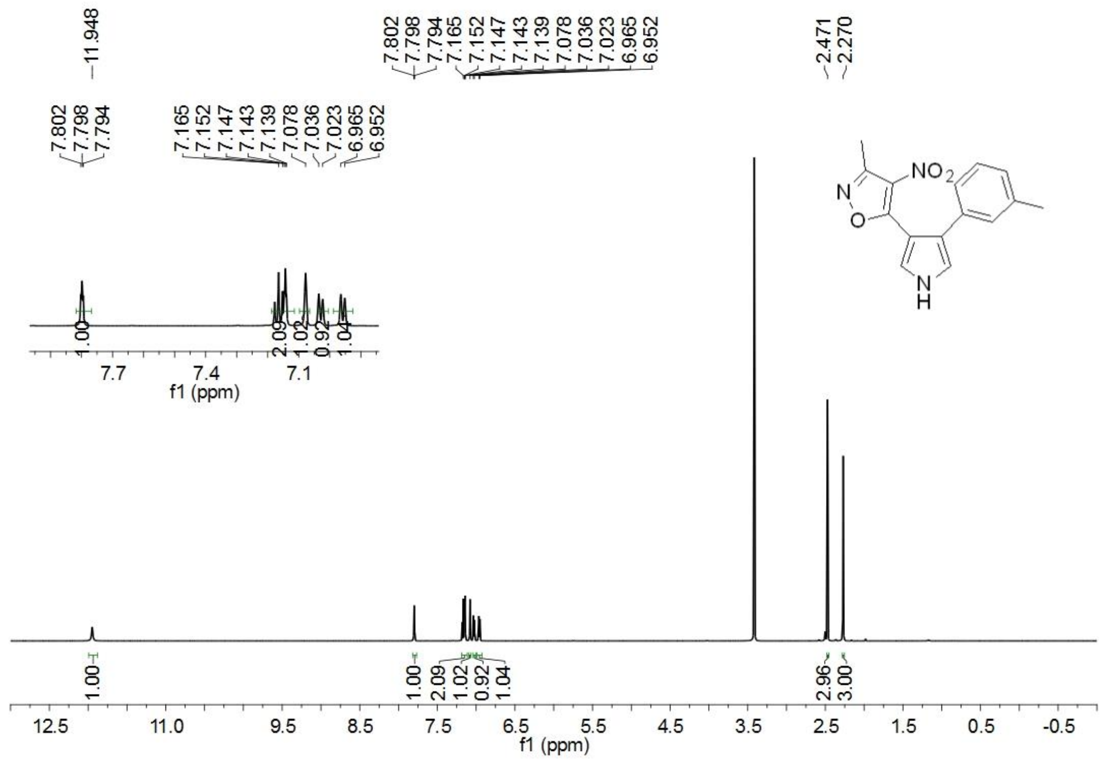

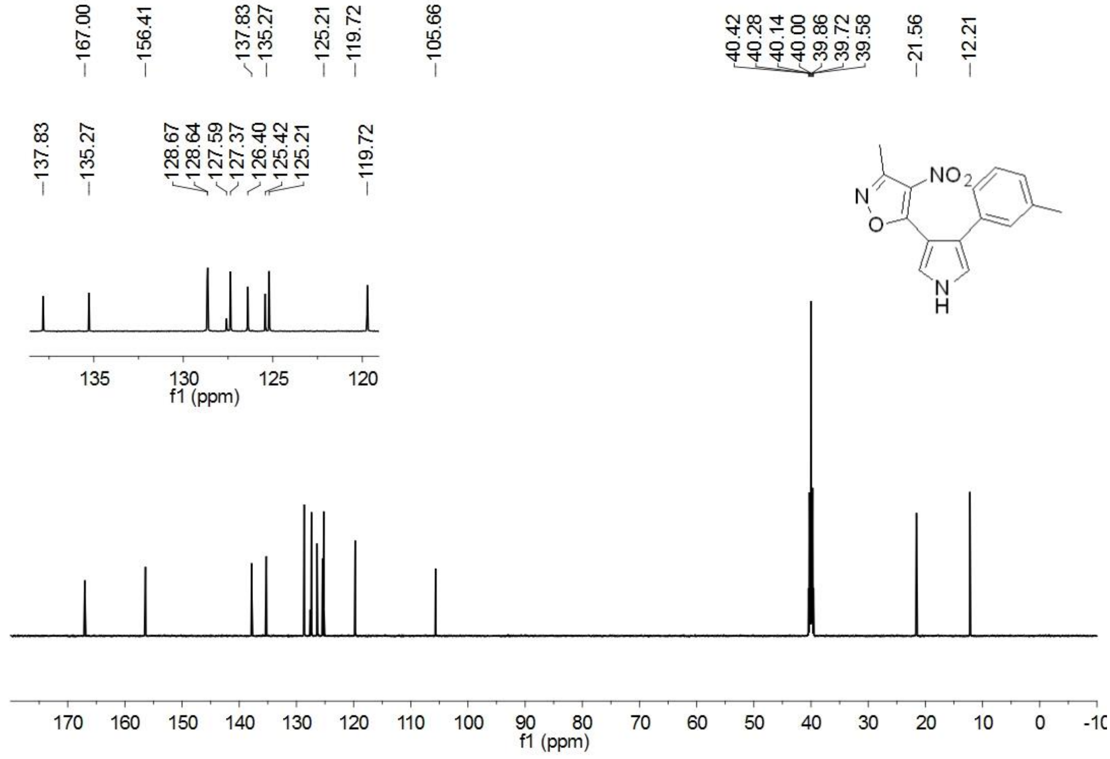

^{1}H NMR and ^{13}C NMR of **3ag**

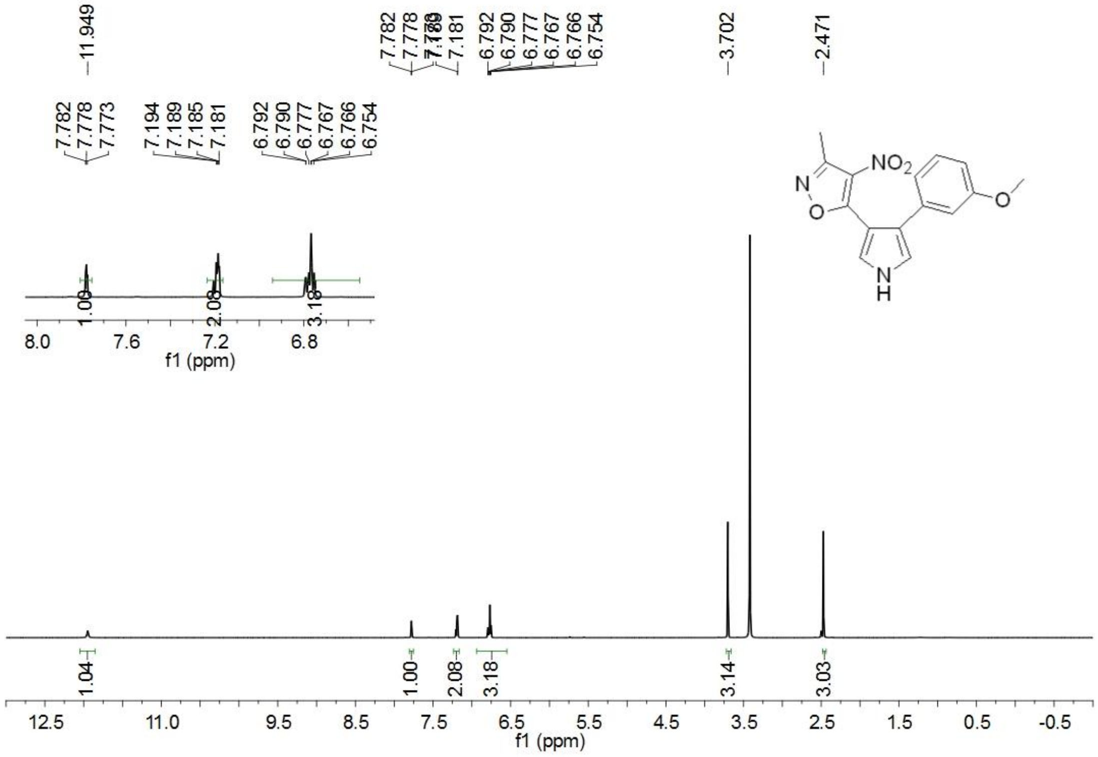

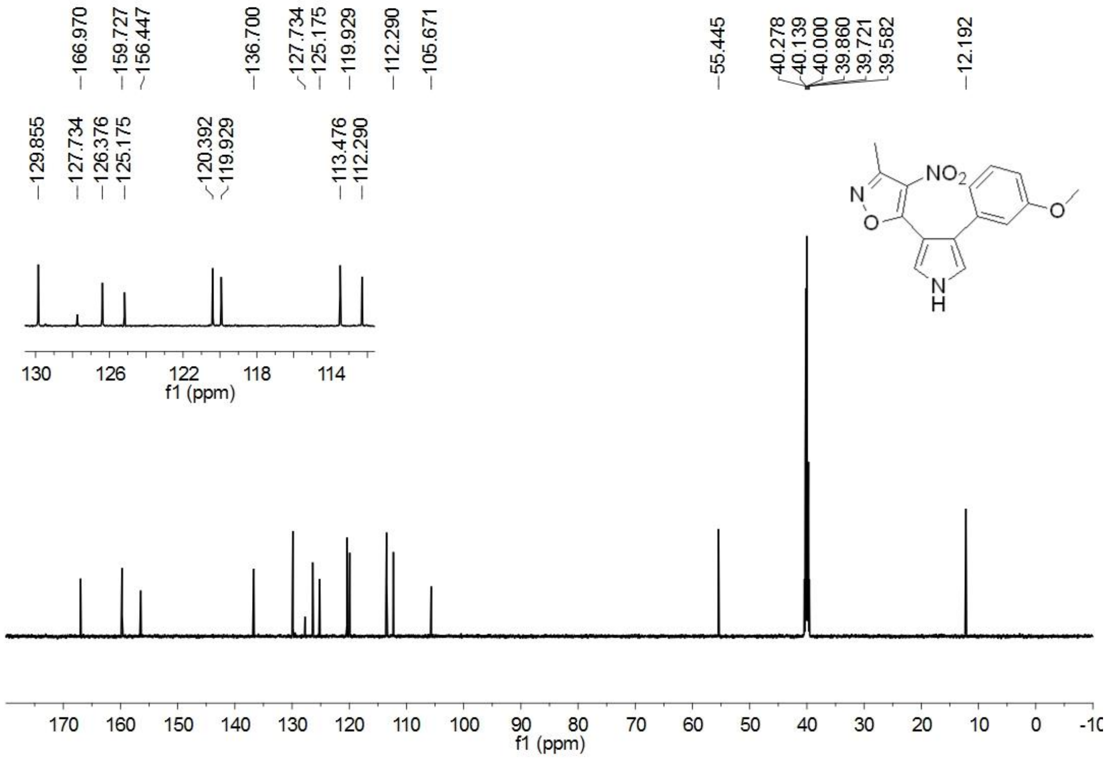

^{1}H NMR and ^{13}C NMR of **3ah**

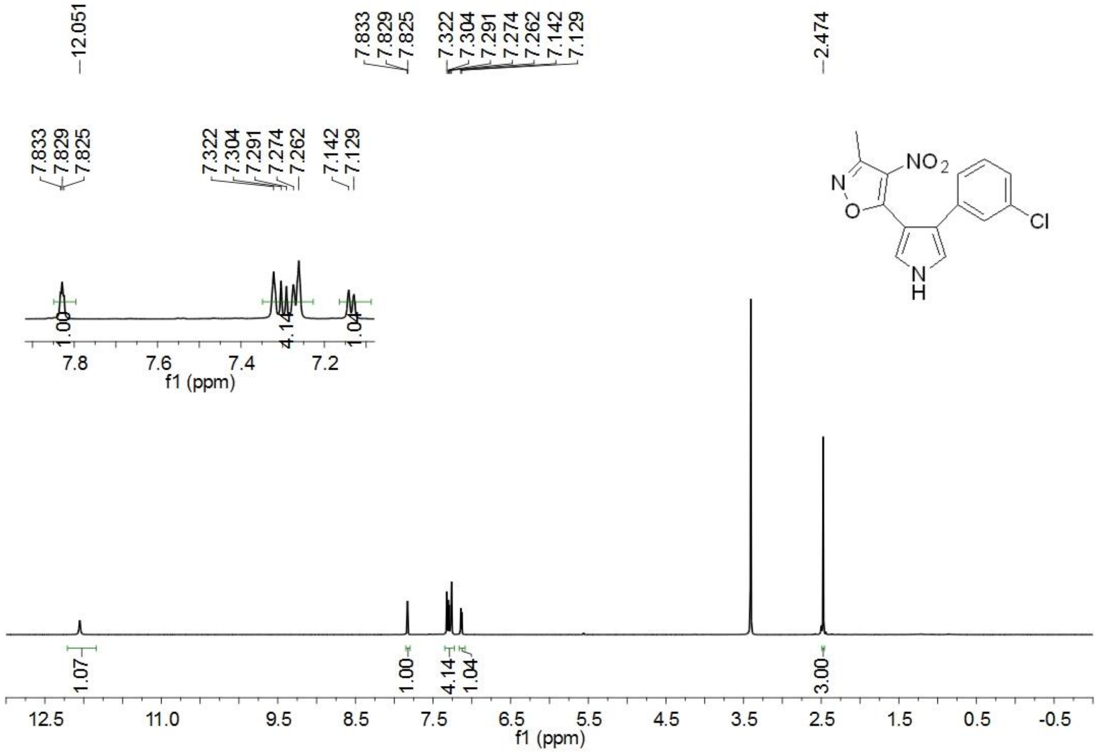

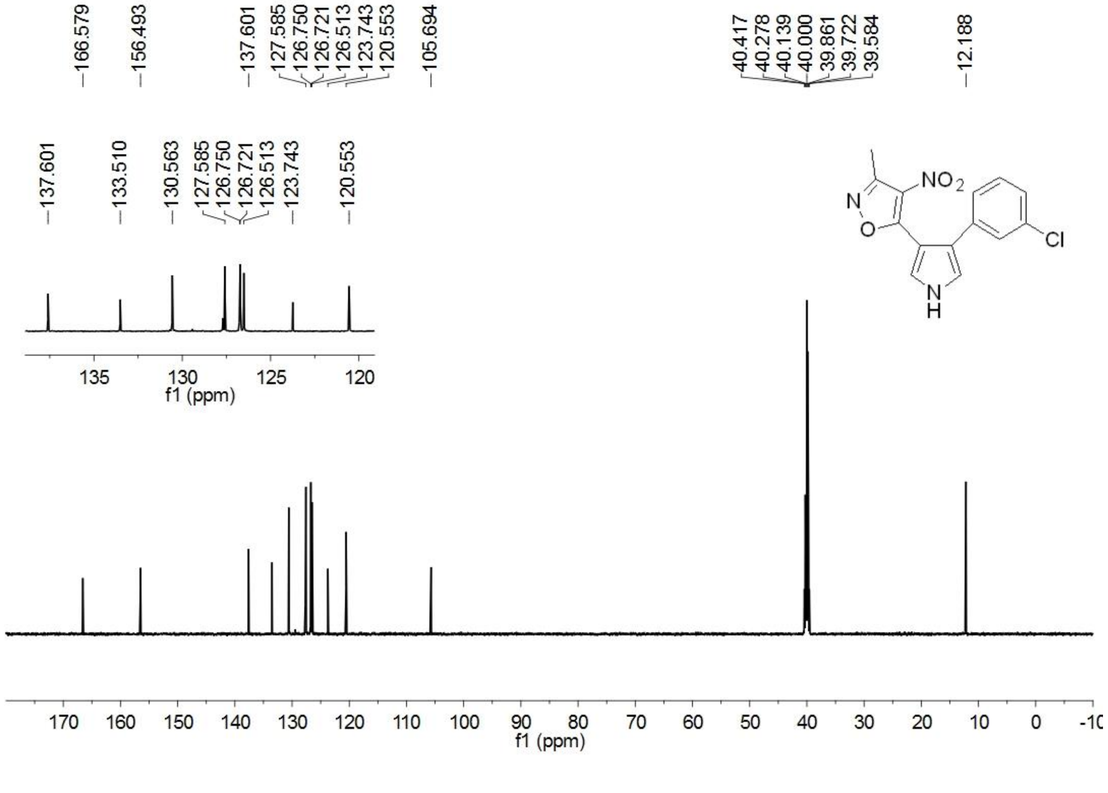

^{1}H NMR and ^{13}C NMR of **3ai**

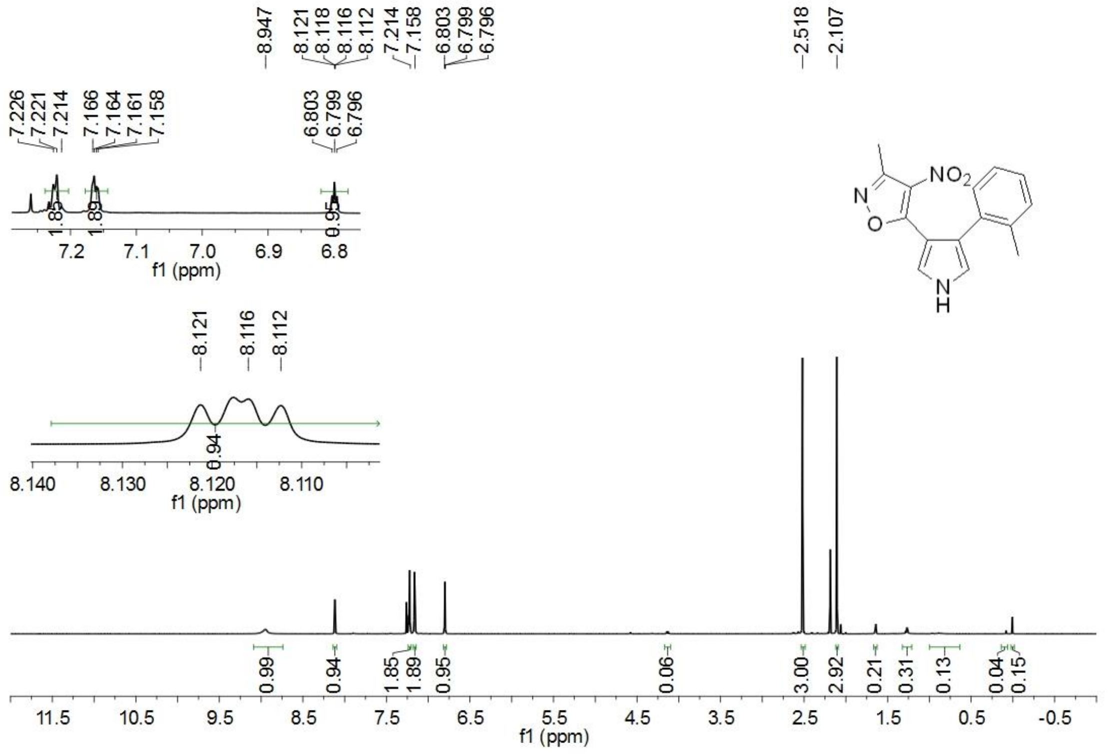

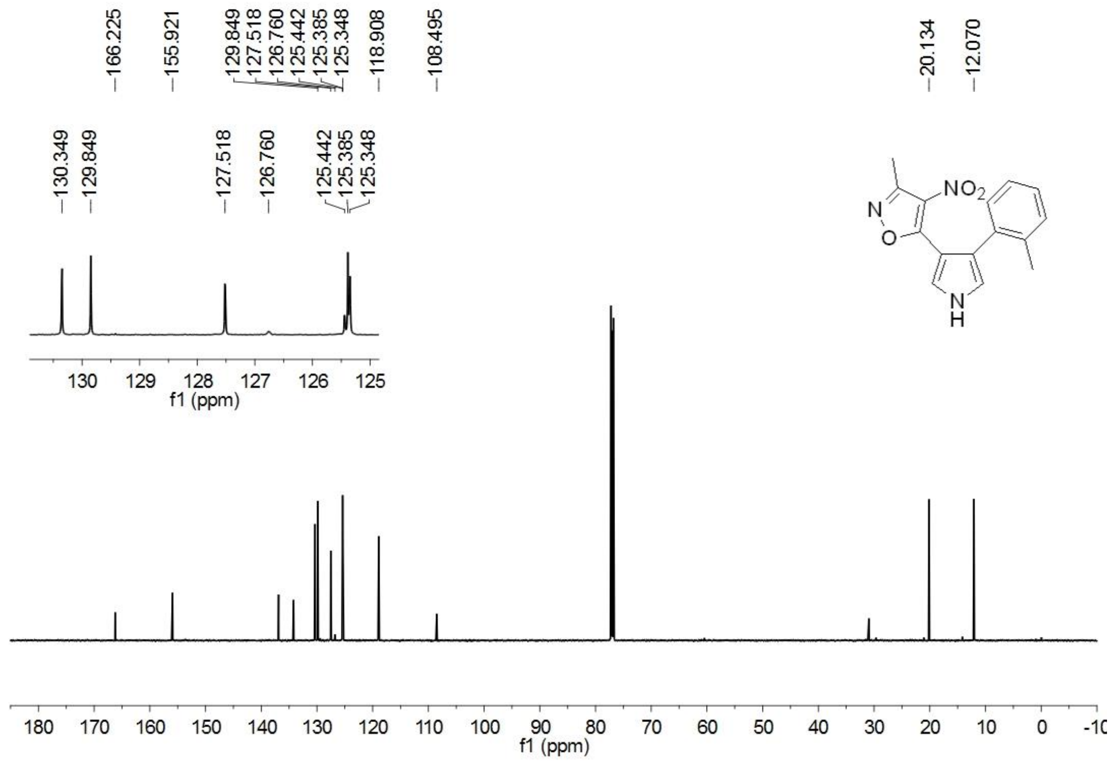

^{1}H NMR and ^{13}C NMR of **3aj**

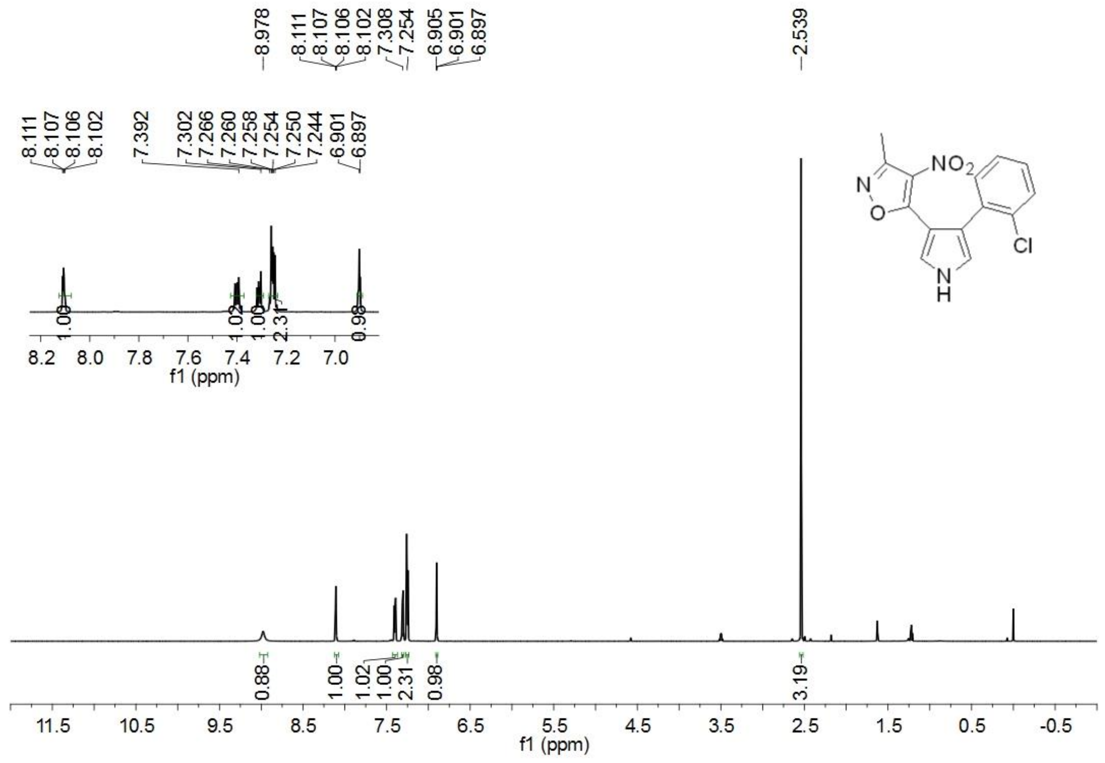

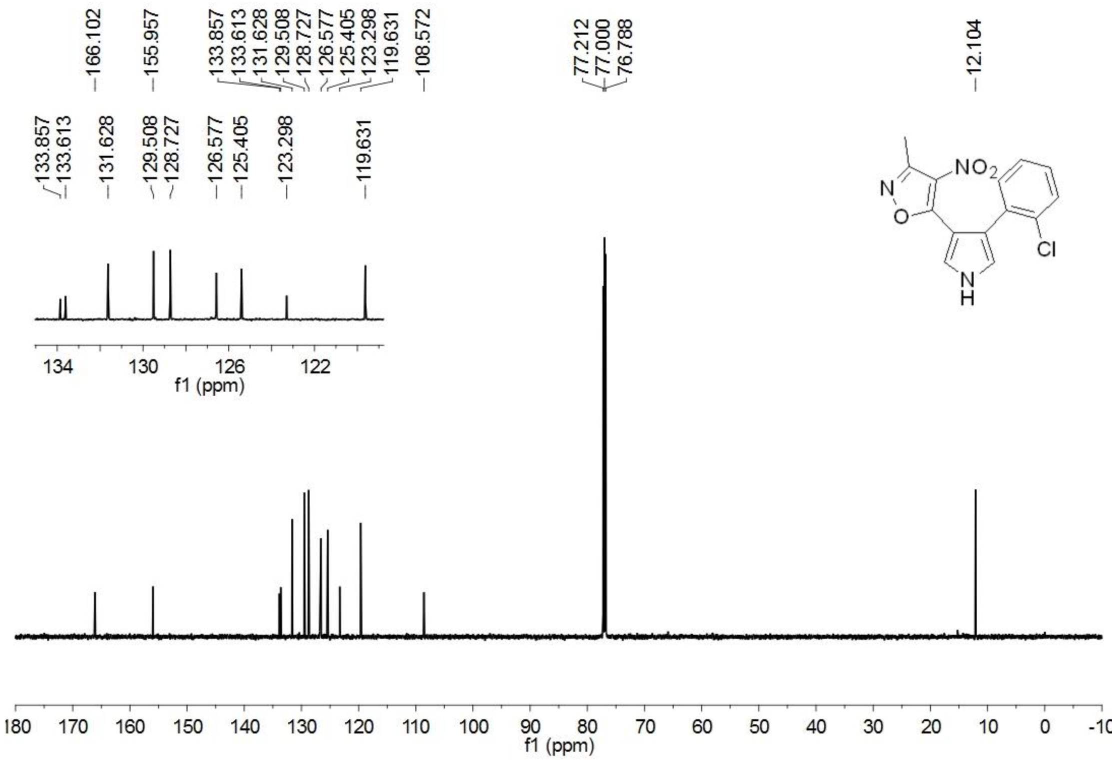

^{1}H NMR and ^{13}C NMR of **3ak**

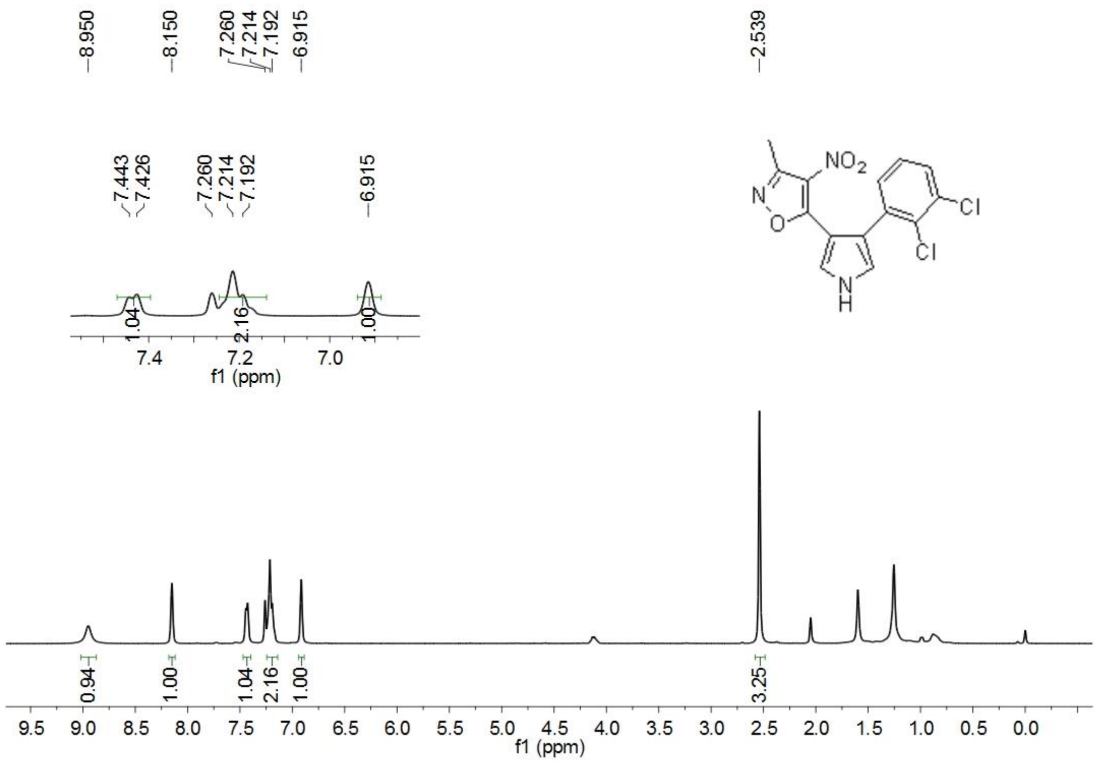

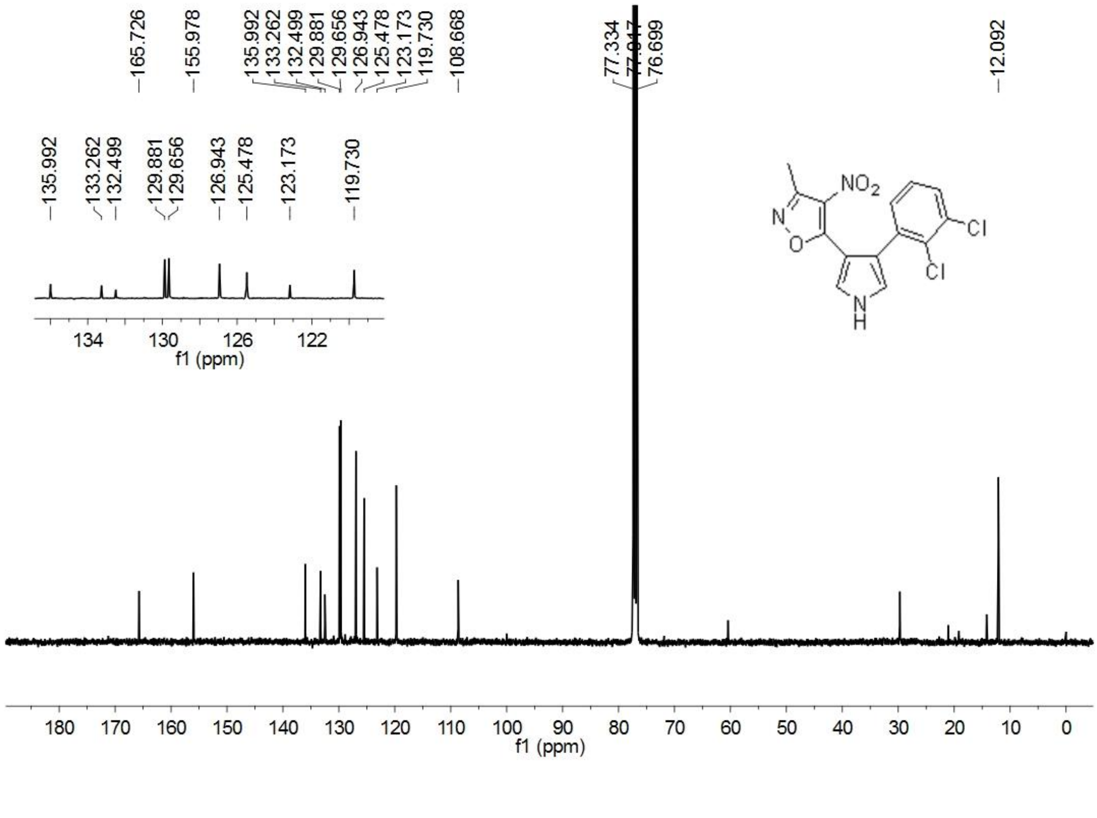

^{1}H NMR and ^{13}C NMR of **3al**

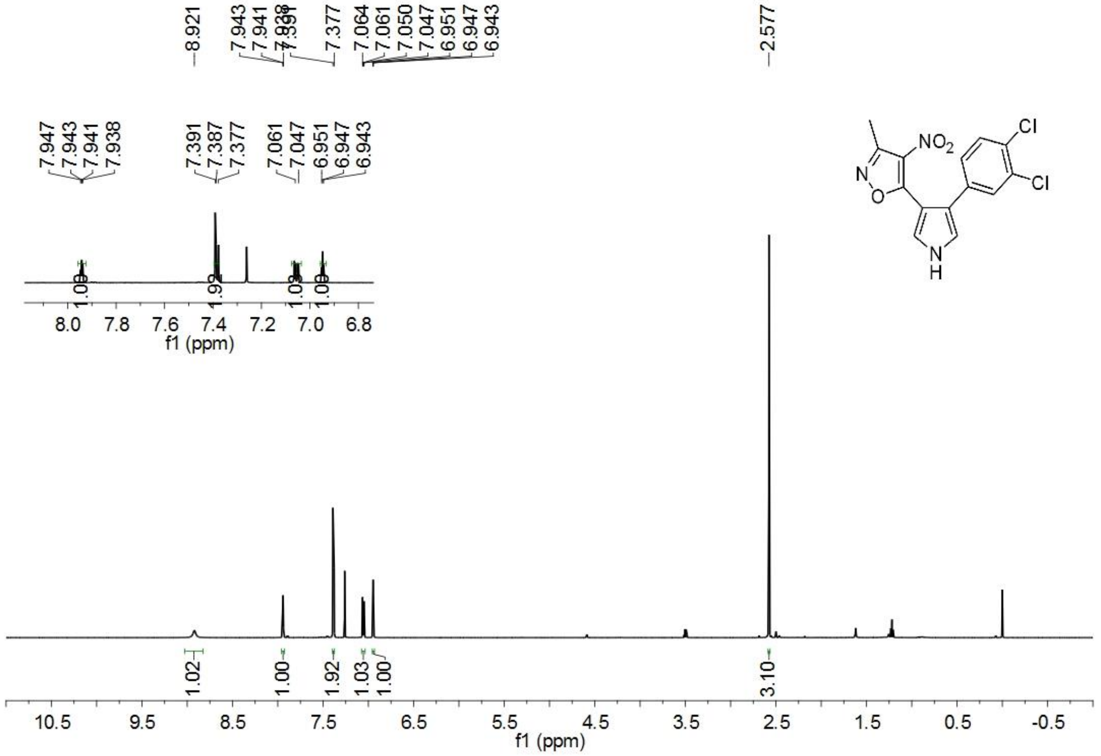

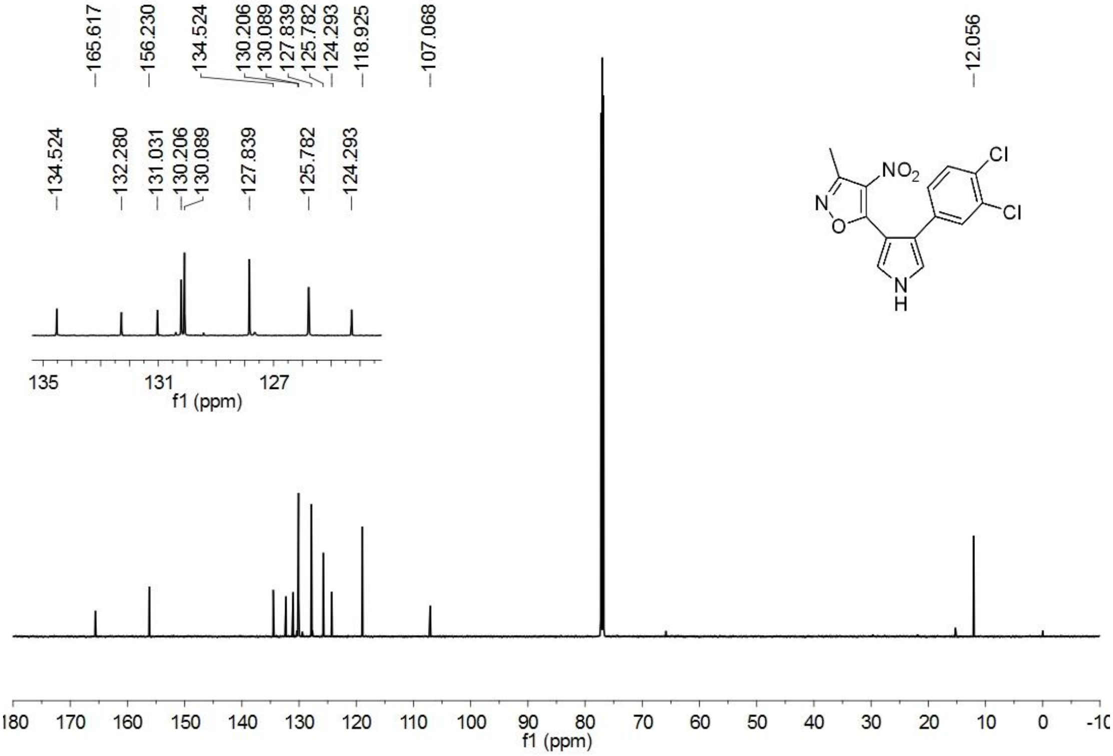

^{1}H NMR and ^{13}C NMR of **3am**

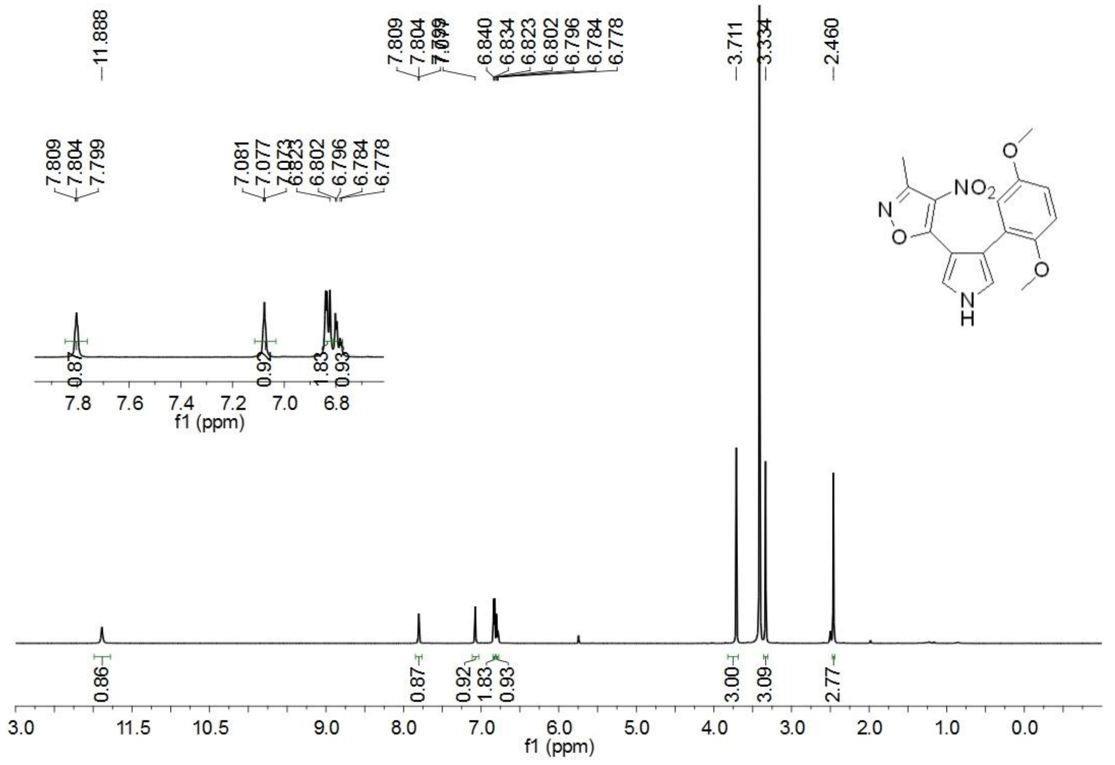

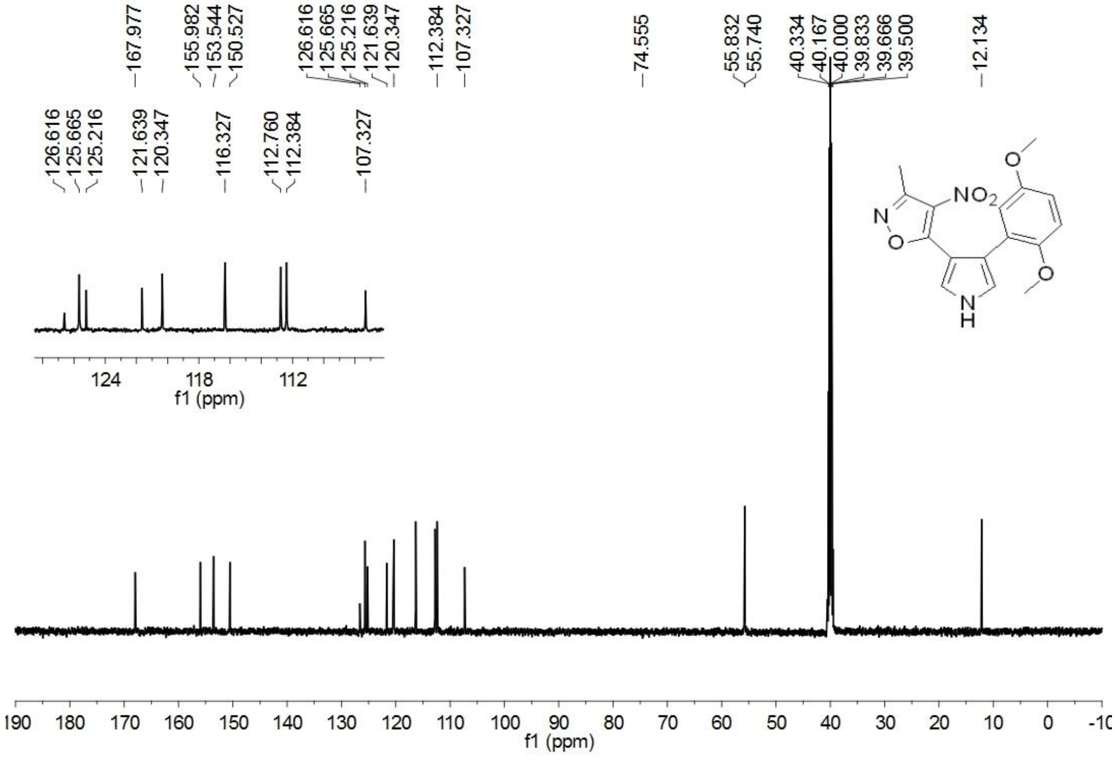

^{1}H NMR and ^{13}C NMR of **3an**

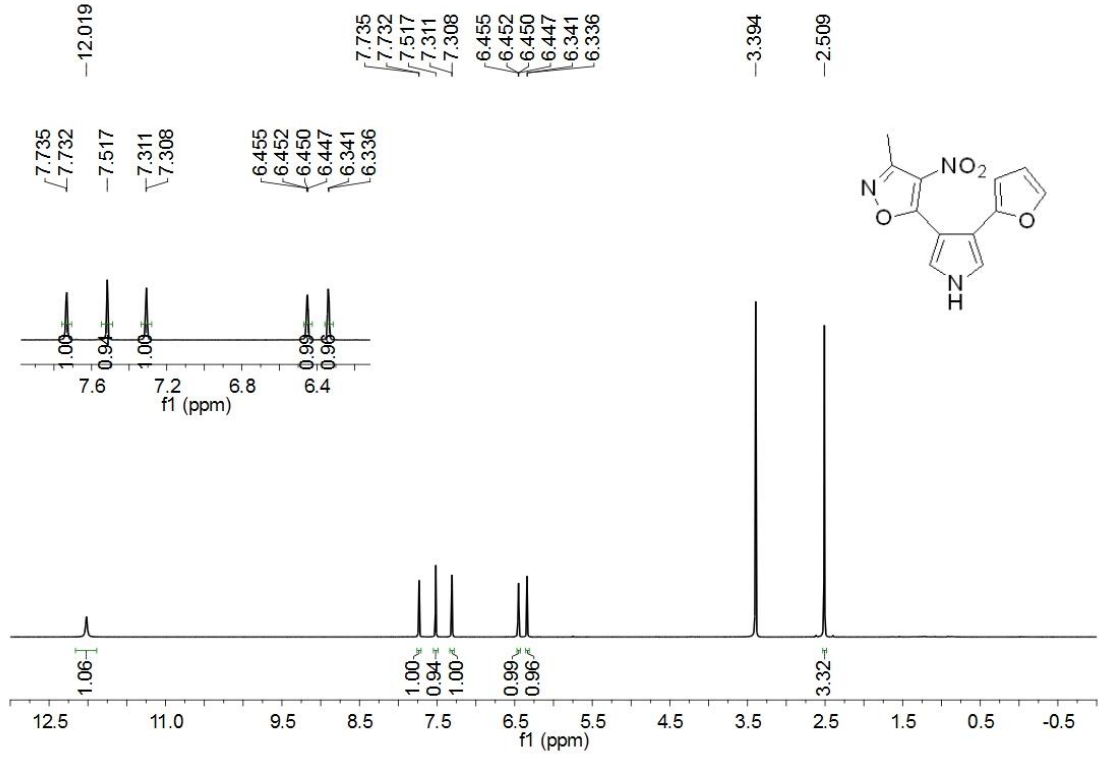

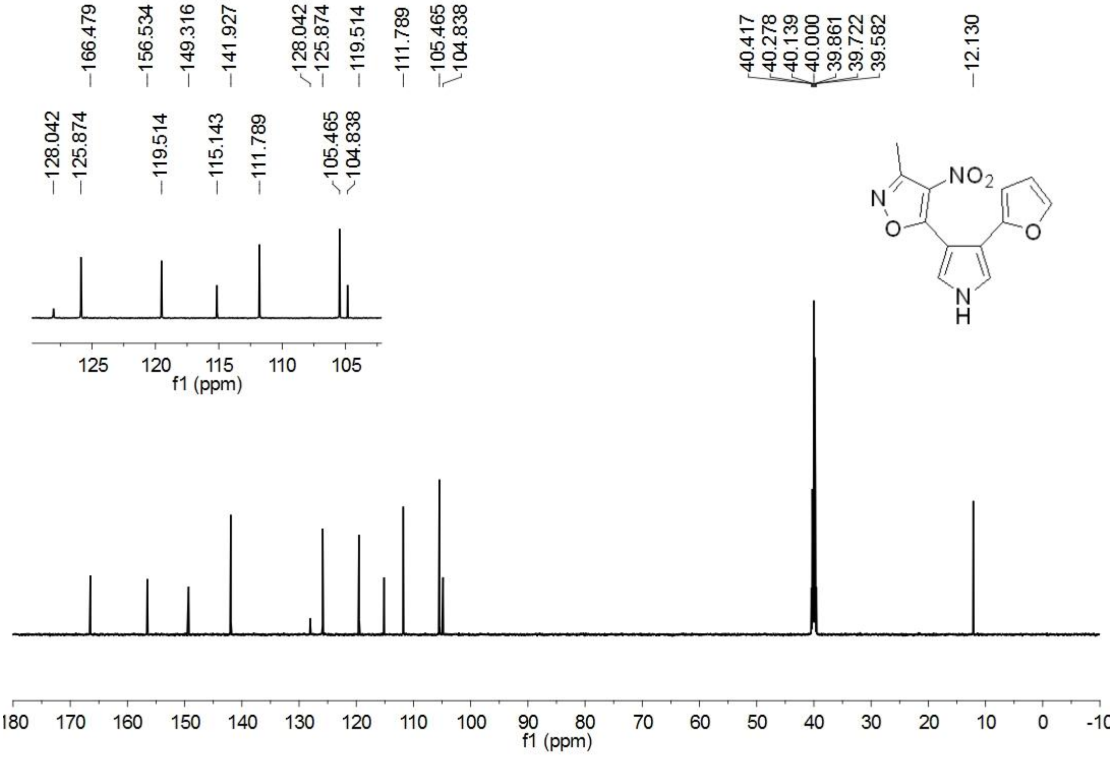

^{1}H NMR and ^{13}C NMR of **3ao**

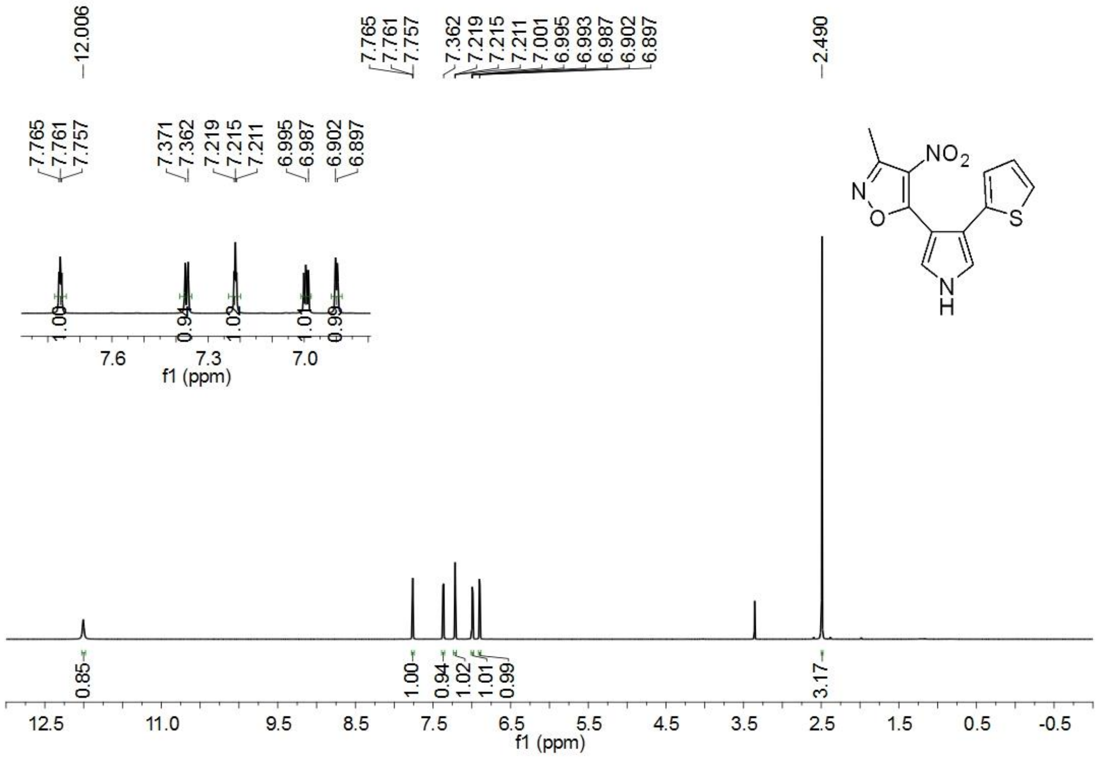

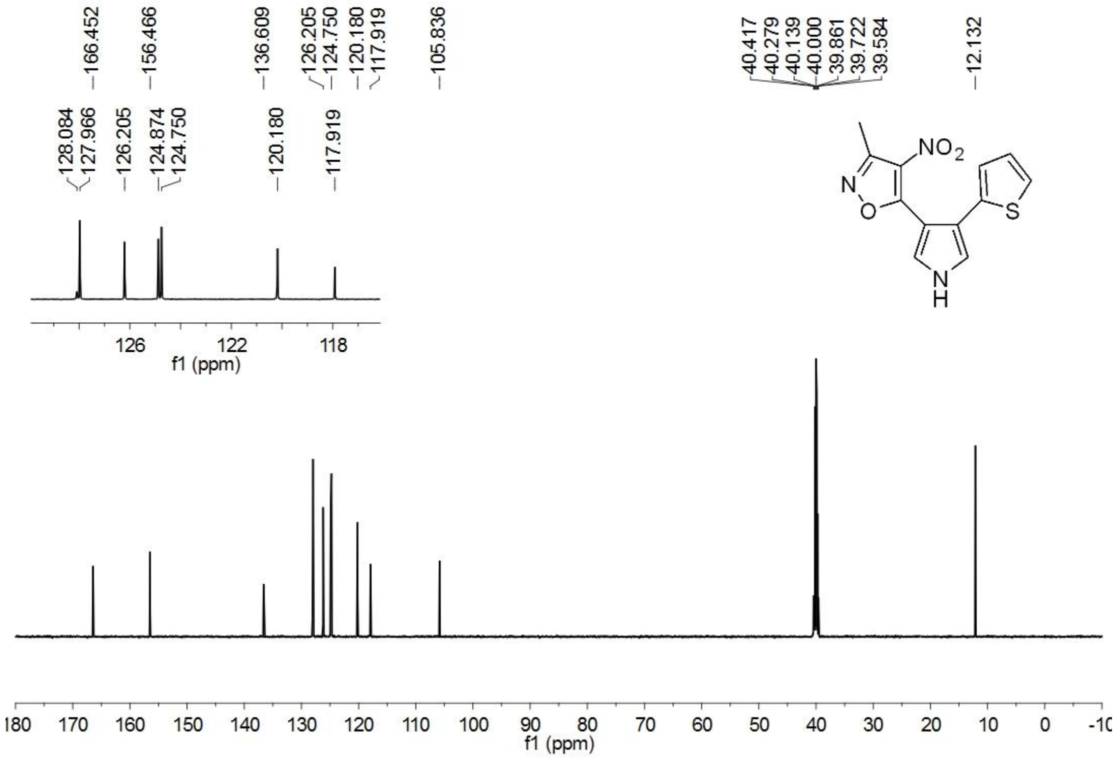

^{1}H NMR and ^{13}C NMR of **3ap**

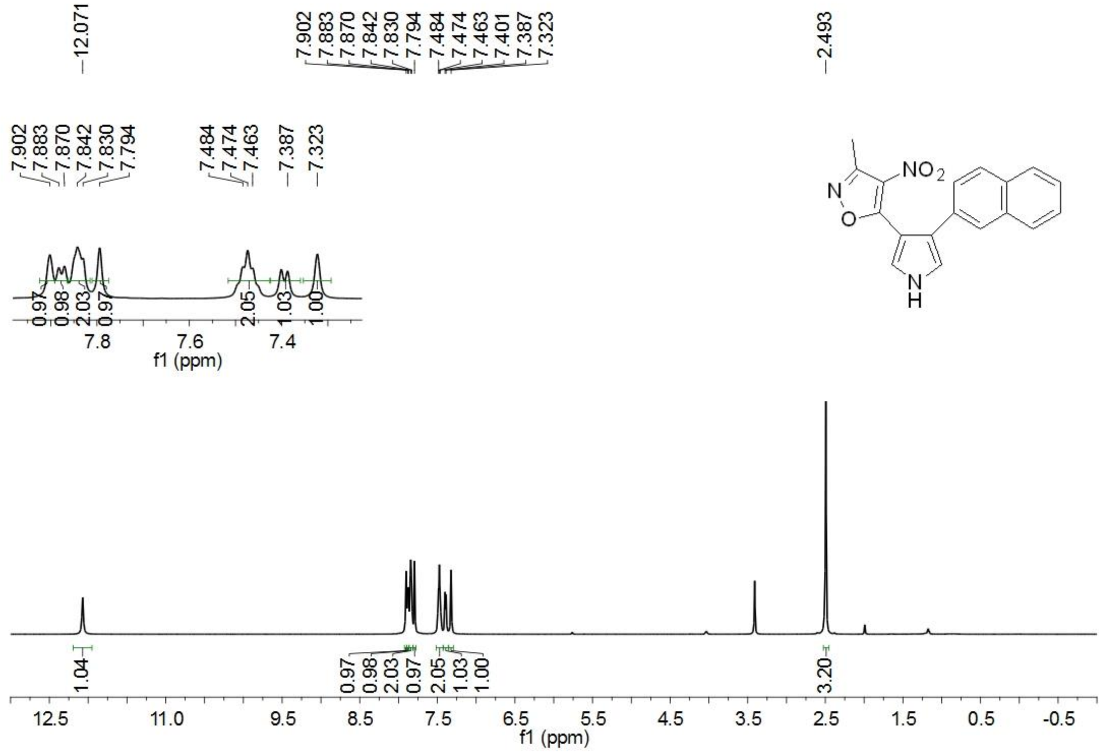

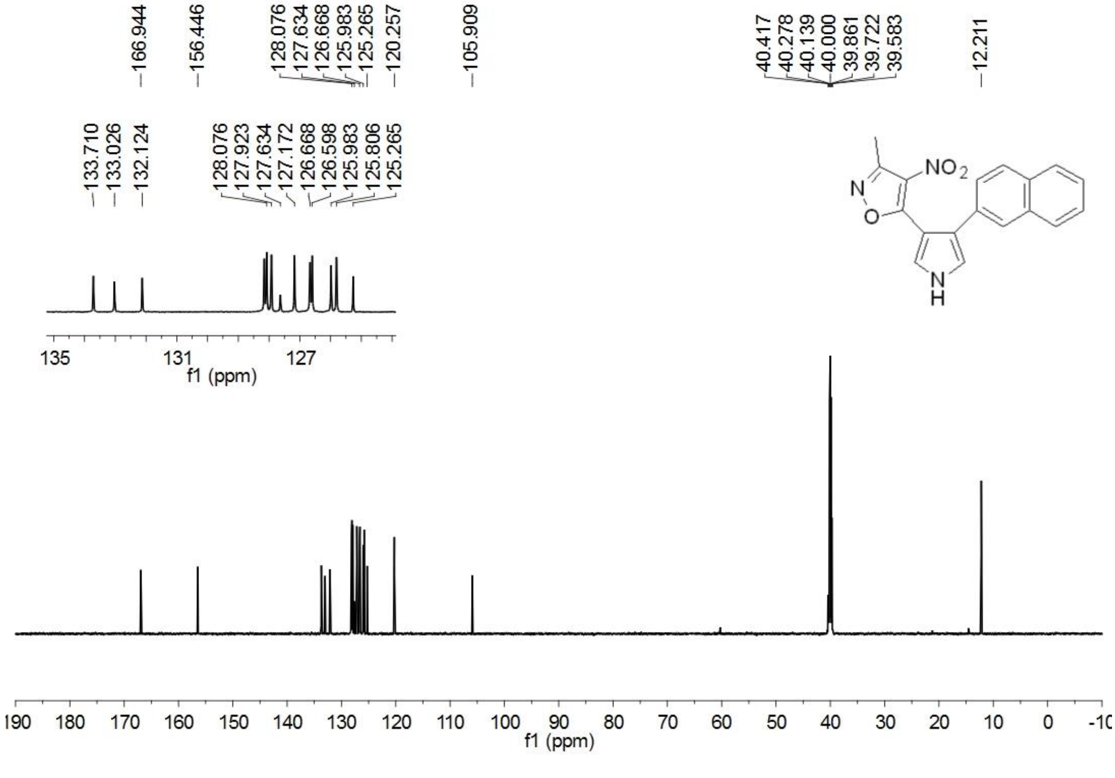

^{1}H NMR and ^{13}C NMR of **3aq**

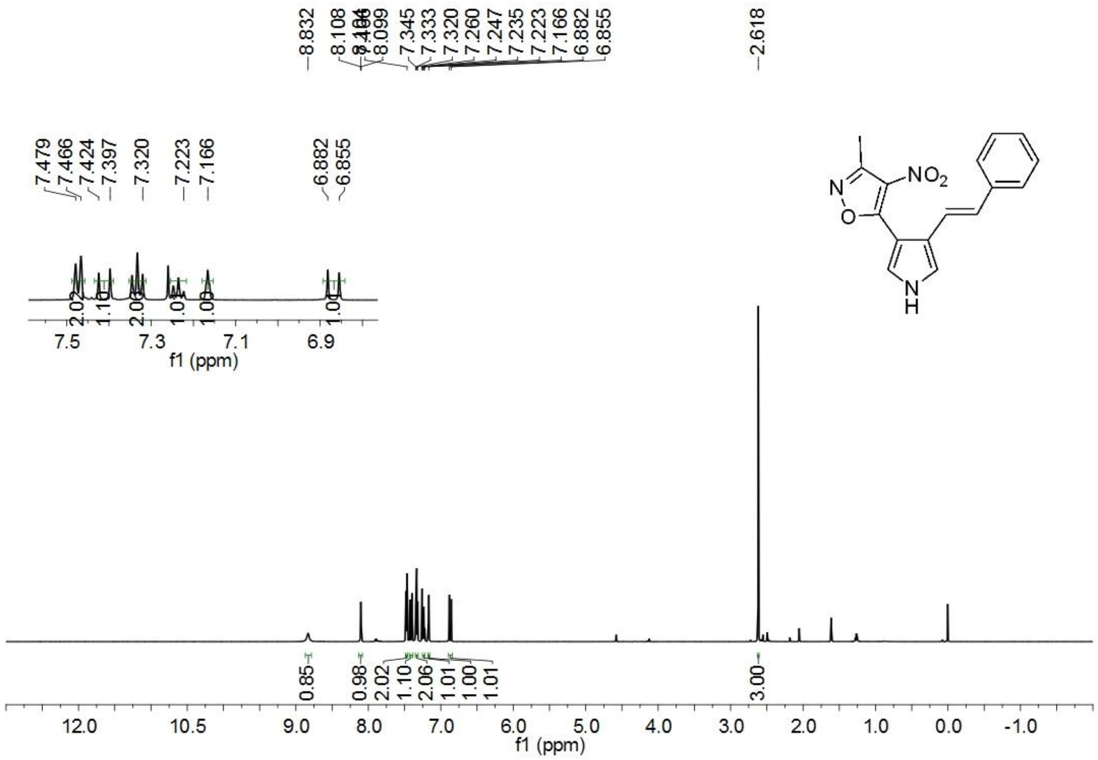

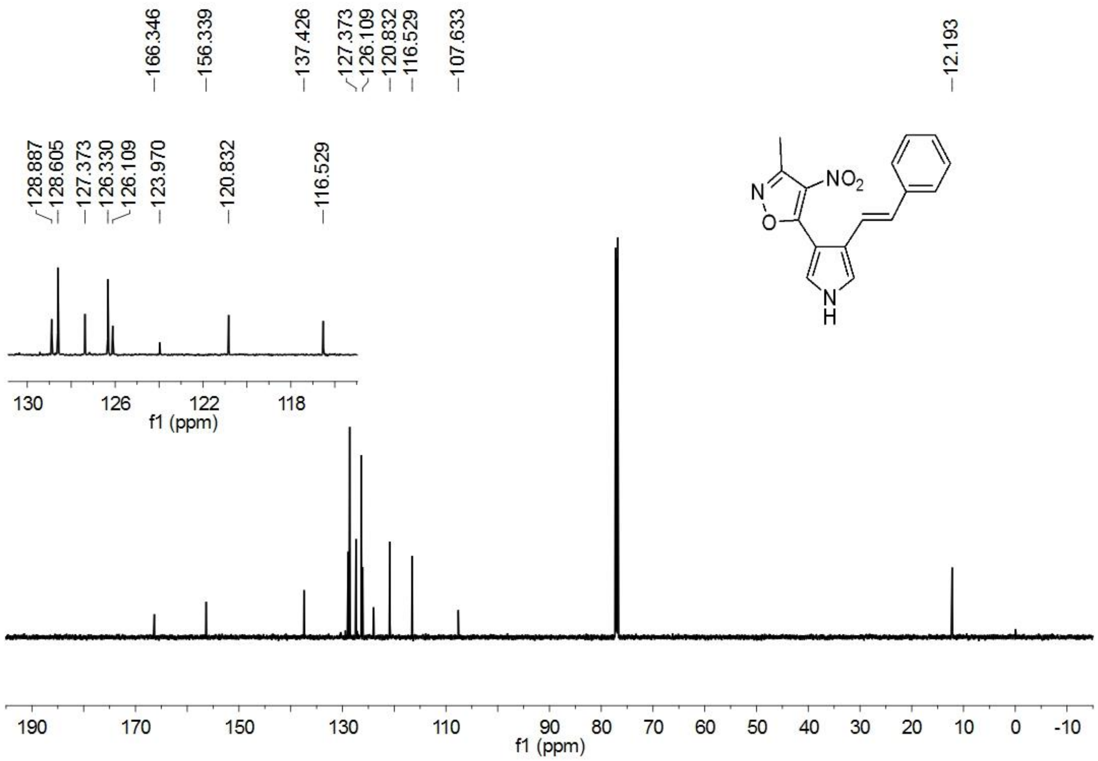

^{1}H NMR and ^{13}C NMR of **3bb**

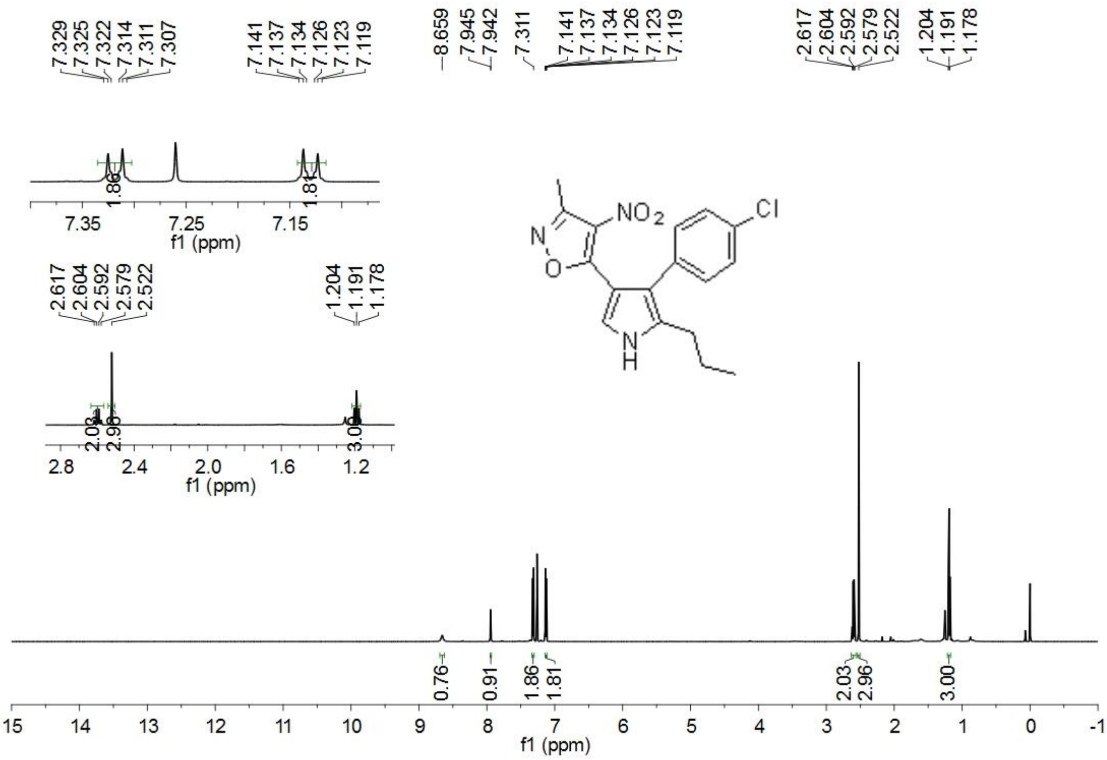

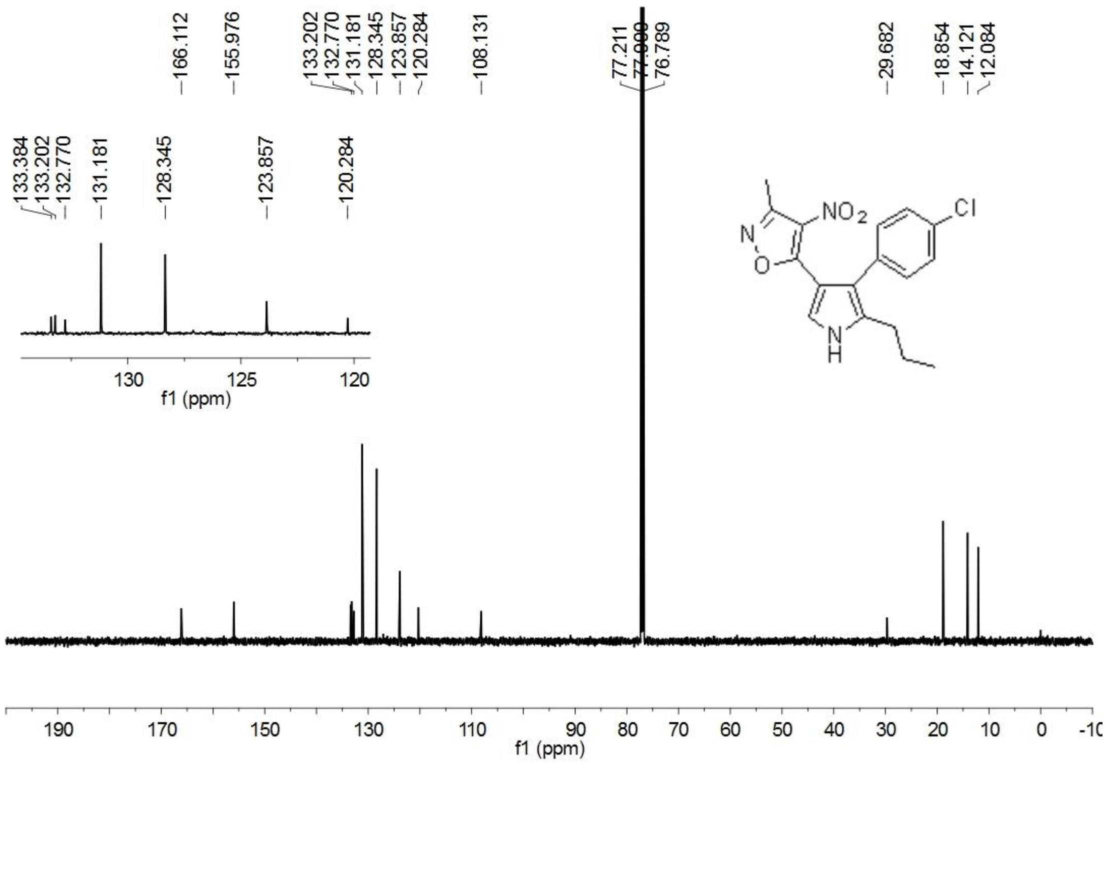

^{1}H NMR and ^{13}C NMR of **3cb**

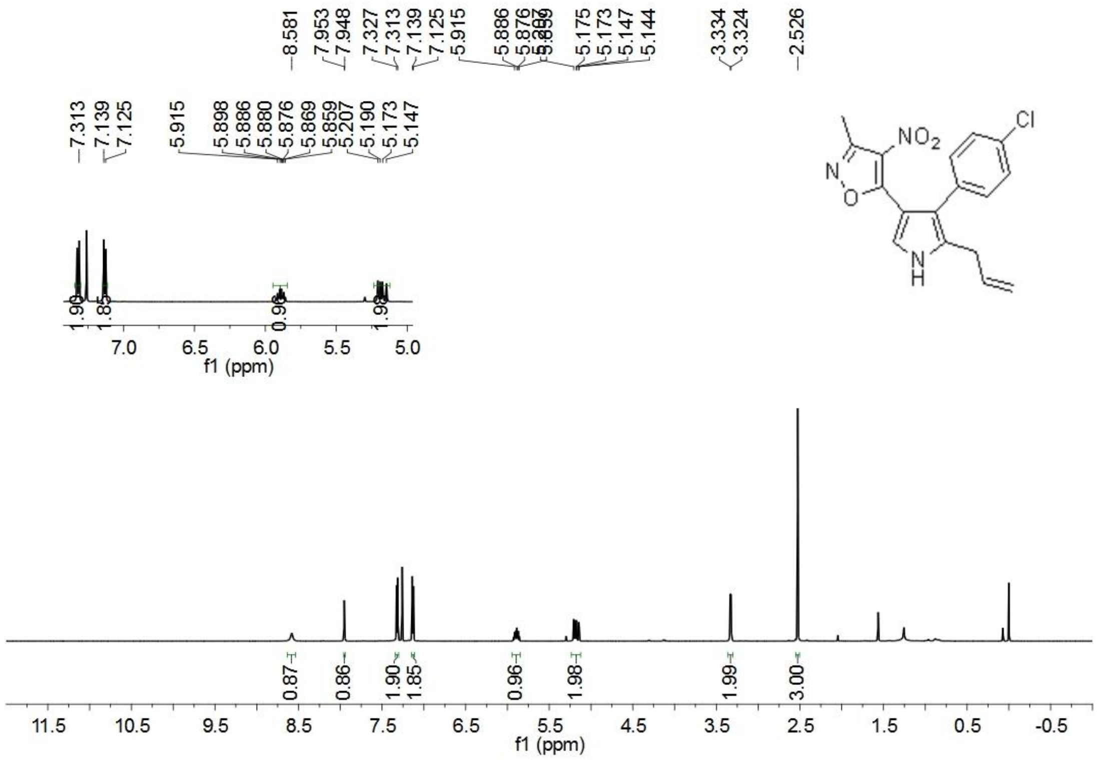

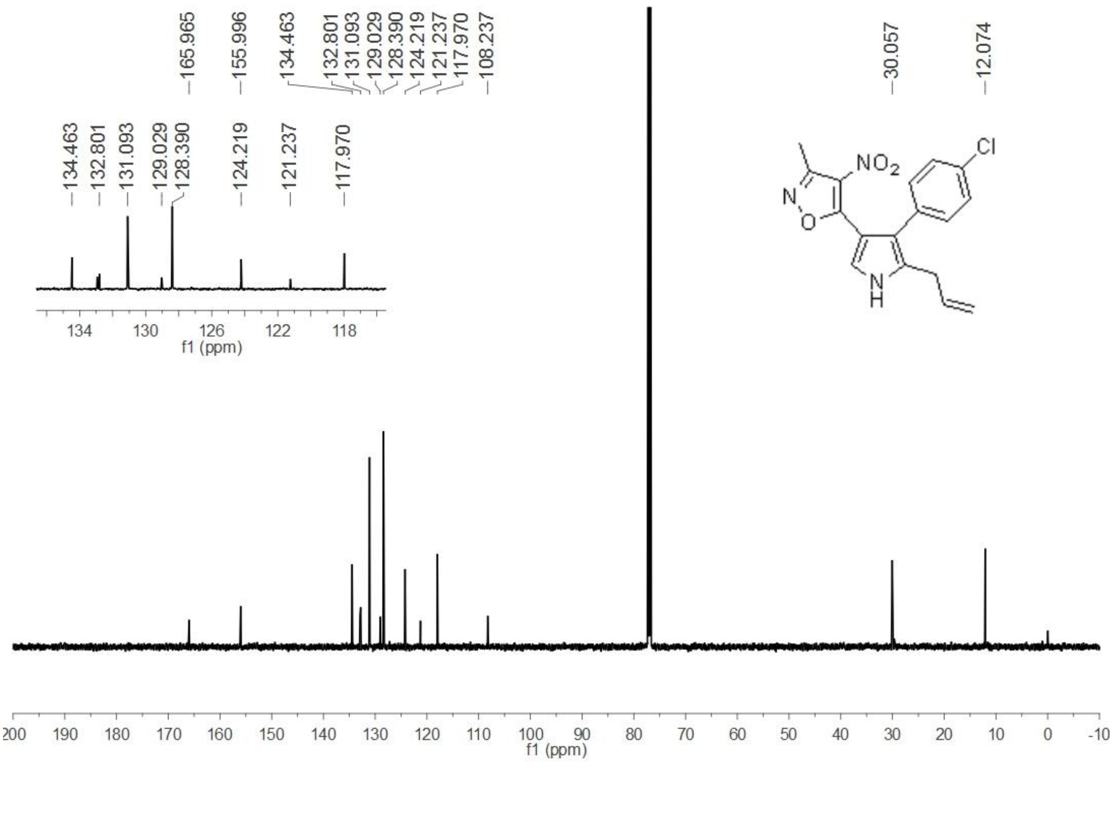

^{1}H NMR and ^{13}C NMR of **3db**

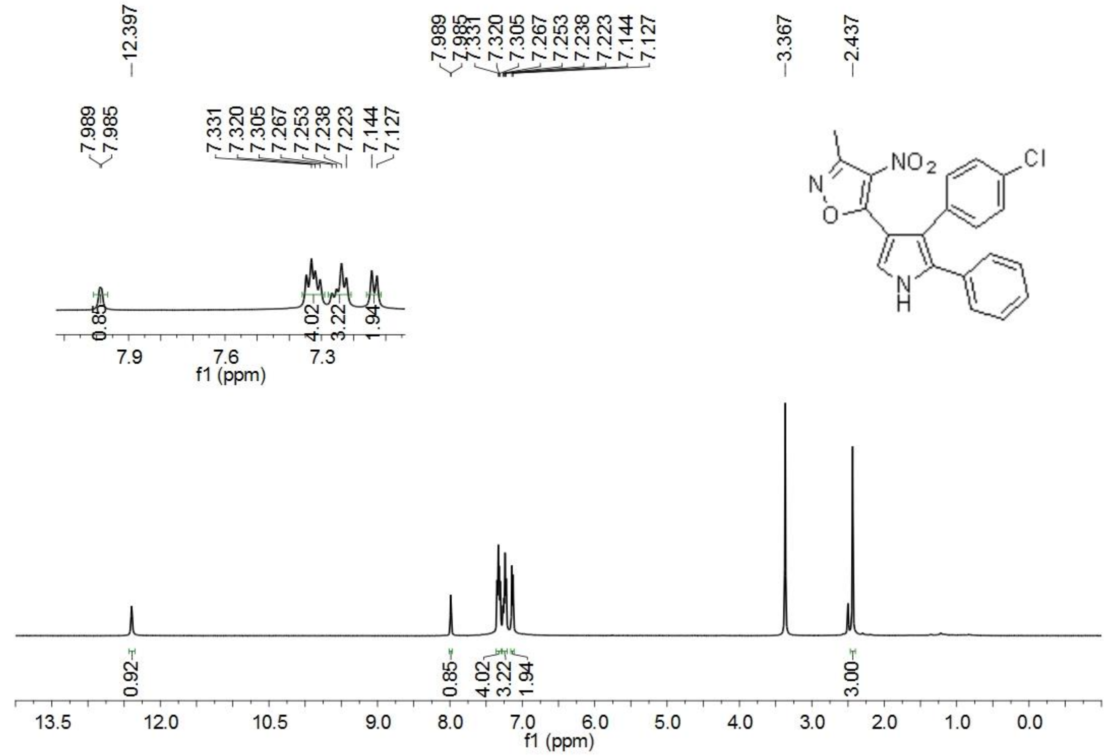

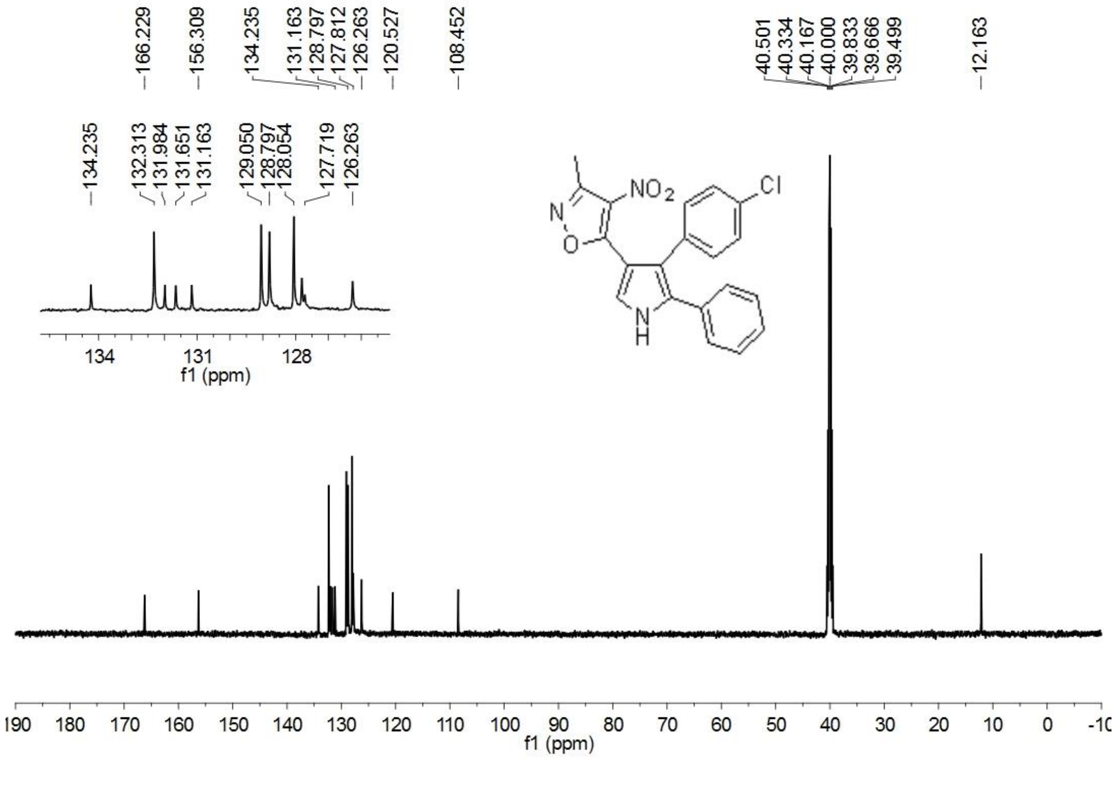

^{1}H NMR and ^{13}C NMR of **3eb**

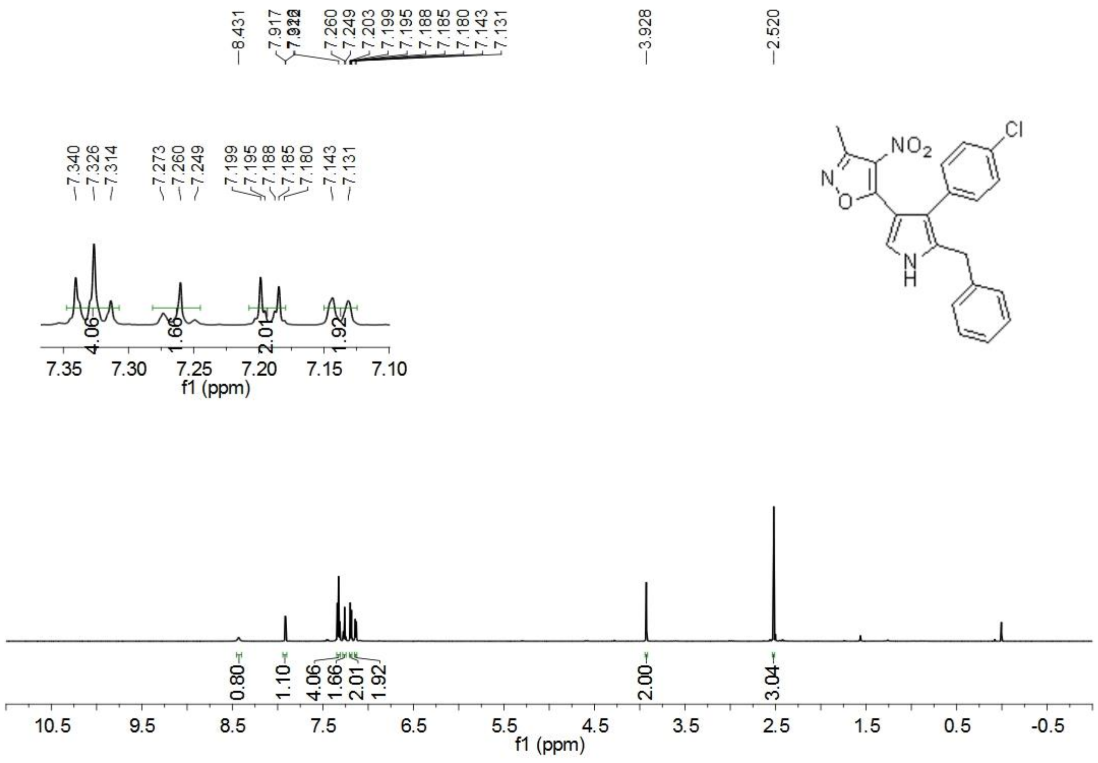

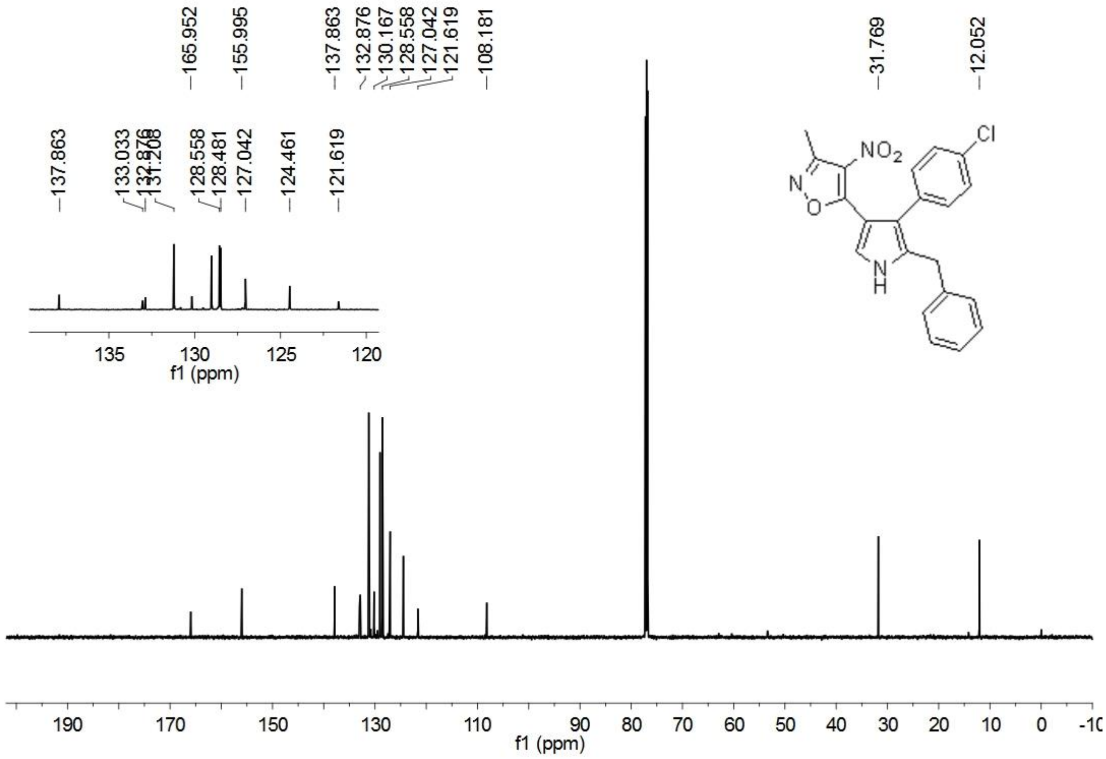

^{1}H NMR and ^{13}C NMR of **3fb**

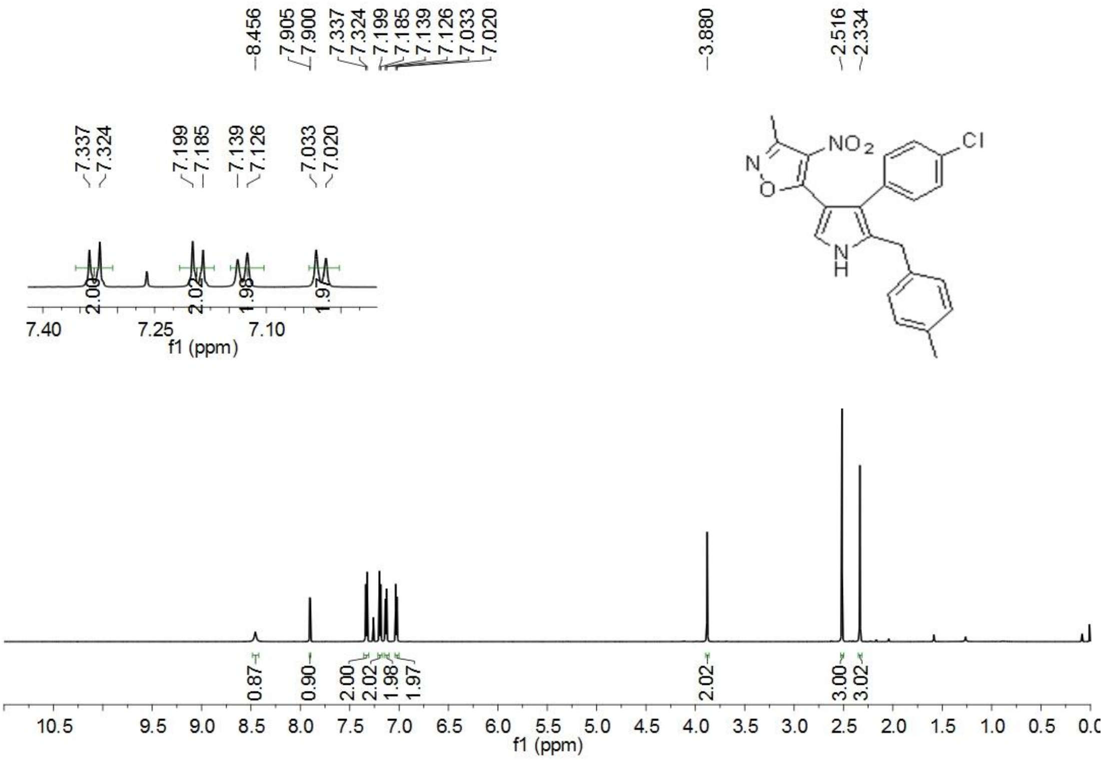

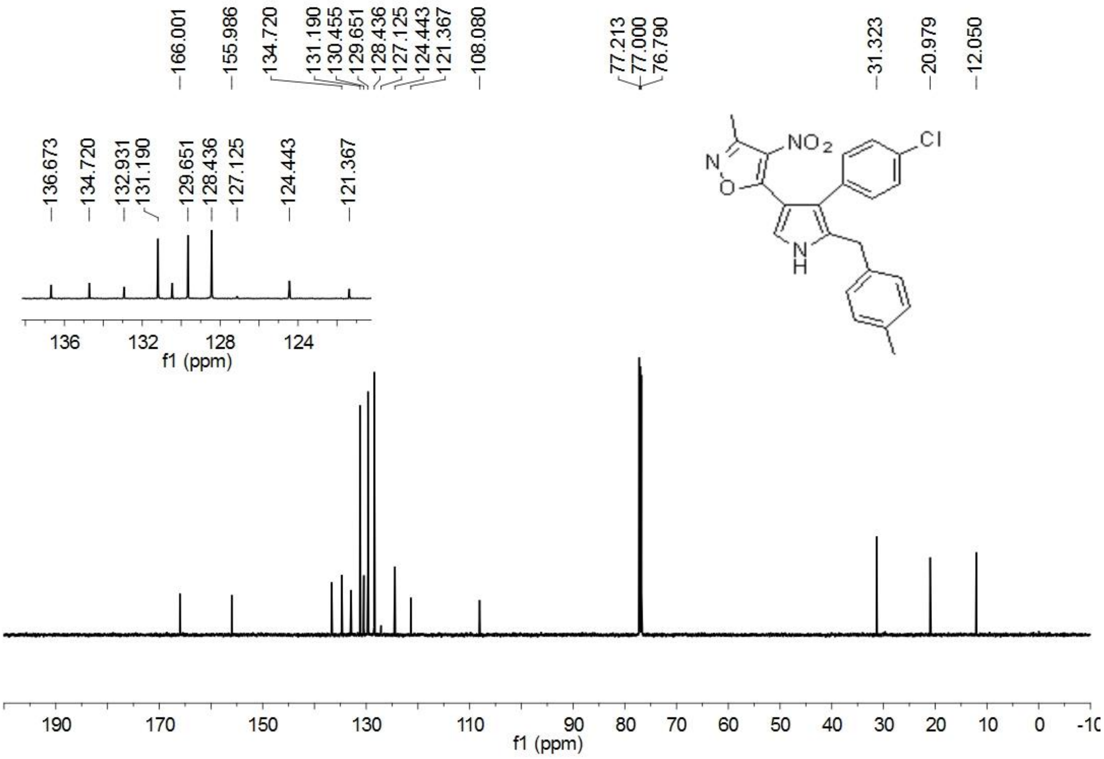

II. Copies of NOE-NMR spectra of compounds **3aq**

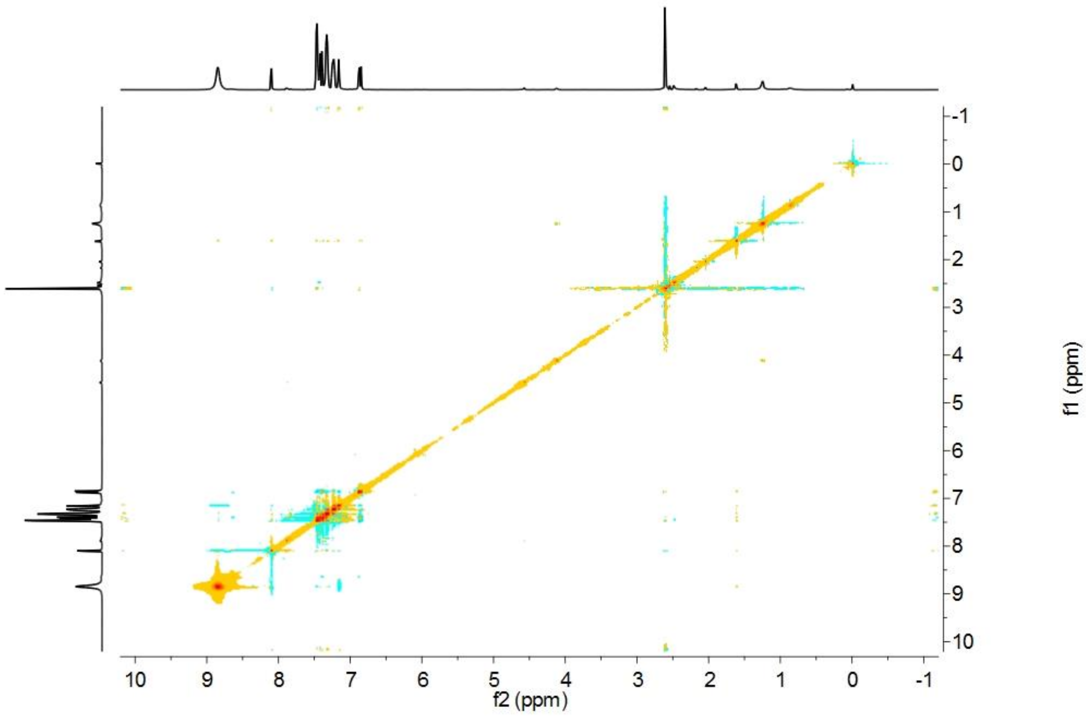

III. Crystal data and structural refinement for **3ac**

1. ORTEP drawing of compound **3ac.**

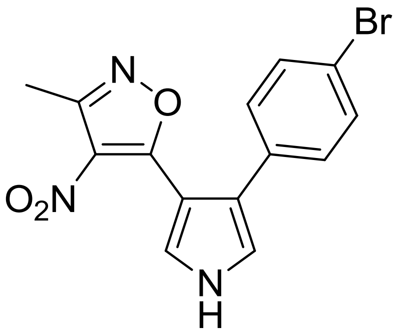

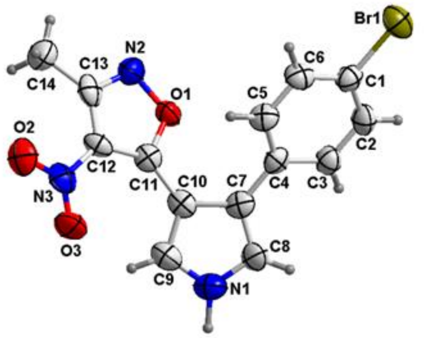

2. Crystal data for **3ac**

$C_{14}H_{10}BrN_3O_3$, Green solid, Mr = 348.15, monoclinic, space group C2/c, a =7.6080(11), b = 7.7910(11), c = 23.078(3) Å, α =90.000, β = 90.000, γ = 90.000 °, V = 1367.9(3) Å, Z = 4, calcd = 1.691, T = 293(2) K, 3166 reflections (2578 unique), 253 refined parameters, R =0.0653 (2578 data with I > 2σ(I)), wR2 = 0.1516. The hydrogen atoms were refined as rigid groups. Crystallographic data for the structures **3ac** have been deposited in the Cambridge Crystallography Data Centre (CCDC No. 1552332).
